# A Review of THz Modulators with Dynamic Tunable Metasurfaces

**DOI:** 10.3390/nano9070965

**Published:** 2019-07-01

**Authors:** Lan Wang, Yaxin Zhang, Xiaoqing Guo, Ting Chen, Huajie Liang, Xiaolin Hao, Xu Hou, Wei Kou, Yuncheng Zhao, Tianchi Zhou, Shixiong Liang, Ziqiang Yang

**Affiliations:** 1 Terahertz Science Cooperative Innovation Center, School of Electronic Science and Engineering, University of Electronic Science and Technology of China, Chendu 610054, China; 2 National Key Laboratory of Application Specific Integrated Circuit, Hebei Semiconductor Research Institute, Shijiazhuang 050051, China

**Keywords:** terahertz modulator, semiconductor material, metasurface

## Abstract

Terahertz (THz) radiation has received much attention during the past few decades for its potential applications in various fields, such as spectroscopy, imaging, and wireless communications. To use terahertz waves for data transmission in different application systems, the efficient and rapid modulation of terahertz waves is required and has become an in-depth research topic. Since the turn of the century, research on metasurfaces has rapidly developed, and the scope of novel functions and operating frequency ranges has been substantially expanded, especially in the terahertz range. The combination of metasurfaces and semiconductors has facilitated both new opportunities for the development of dynamic THz functional devices and significant achievements in THz modulators. This paper provides an overview of THz modulators based on different kinds of dynamic tunable metasurfaces combined with semiconductors, two-dimensional electron gas heterostructures, superconductors, phase-transition materials, graphene, and other 2D material. Based on the overview, a brief discussion with perspectives will be presented. We hope that this review will help more researchers learn about the recent developments and challenges of THz modulators and contribute to this field.

## 1. Introduction

Terahertz (THz) frequencies are located in the range from 0.1 to 10 THz, and the corresponding wavelengths range from 3 mm to 30 µm between the microwave and infrared regions. Due to their high spatial resolution and time resolution [1], terahertz imaging and terahertz spectroscopy constitute two key technologies for terahertz applications. Moreover, compared to X-ray, THz waves cause negligible damage to cells because of their low photon energy and thus can be used for non-invasive detection of organisms and with broader prospects in the medical field. In addition, terahertz technology has been widely used for research on the properties of semiconductor and superconductor materials. More importantly, a transmission rate of more than 100 Gbps can be achieved when utilizing THz waves for data transmission in communication applications. These properties and potential applications make terahertz technology a very important cross-cutting frontier in security checking, wireless communications, imaging, spectroscopy and so on, providing a very attractive opportunity for technological innovation, national economic development and national security.

**Figure 1:**
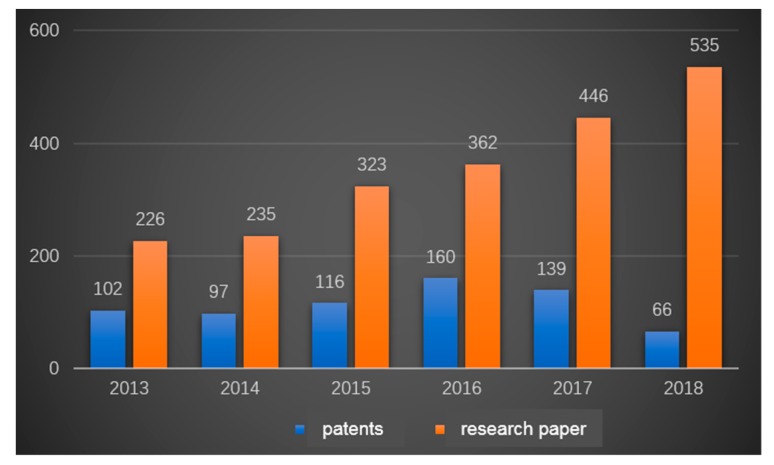
Patents and papers on terahertz applications published annually from 2013 to 2018.

Among the potential THz applications, high-speed imaging and wireless communication are two important research directions that may bring great breakthroughs in modern electronic information technology. Therefore, under this background, the effective and ultra-fast regulation of THz waves makes it possible to apply data transmission in communication and imaging, which is of high demand and has been the subject of intensive research. Furthermore, the breakthrough of terahertz modulators would definitely bring important developments to THz communication technology based on a direct modulation approach. This approach is one of the promising ways to develop long-distance communication and high-speed and high-resolution THz imaging with compressive sensing computational algorithms. However, it is difficult to find a material with a high- speed response to terahertz waves in nature, and the traditional modulation methods are not entirely suitable for the terahertz wave bands. The modulation of THz waves with high speed and high modulation depth is recognized as a bottleneck in this research area. Thus, during the past 10 years, THz modulators have captured worldwide attention. From 2013 to 2018, more than 2000 papers have been presented with different key words related to THz technology, as shown in Figure 1.

**Figure 2:**
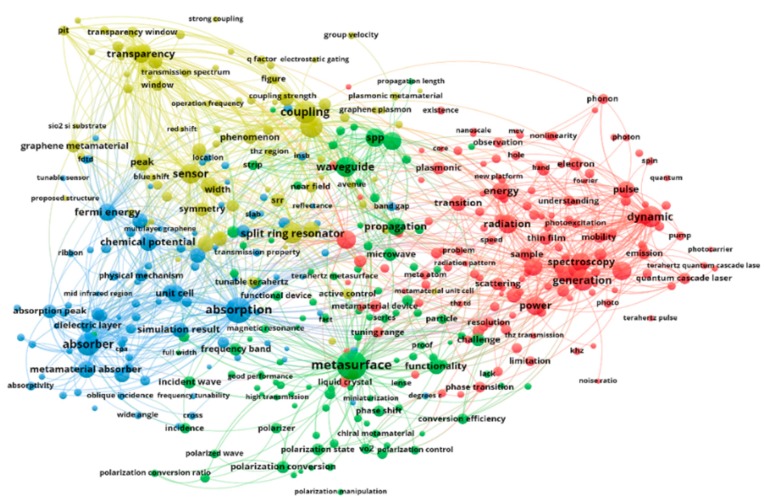
Keyword diagrams in terahertz papers, where the circle size represents the keyword heat.

Among the keywords diagrams in terahertz papers (Figure 2), metasurfaces as a hot keyword, are two-dimensional (2D) versions of metamaterials with subwavelength thicknesses [2], which are usually defined as three-dimensional (3D) artificial nanostructures with exceptional electromagnetic properties. Metasurface designs can be used to develop cloaking, optical vortices [3,4,5], polarization converters [6], etc. On the other hand, the design flexibility of metasurfaces and the improvement in semiconductors provide a promising approach for modulators in practical applications.

Since 2006, terahertz modulators and other active devices have introduced semiconductor materials to metallic metasurface structures to achieve the dynamic control of spatialterahertz waves under external stimuli. Subsequently, different methods, materials and structures have been proposed and presented. The basic principle involves combining the metasurface with doped silicon, phase change materials, ferroelectric thin films, superconducting materials, modulation doped heterojunctions, graphene and so on. The resonant electromagnetic characteristics of the metasurface can be modulated by applying an excitation method such as a temperature change, illumination or an electric field, thereby realizing the manipulation of the terahertz waves. Thus, the THz modulators can be classified by the physical quantity they modulate, e.g., amplitude, phase, spectrum and orbital angular momentum, or by the different excitations employed, e.g., all-optical modulation, electronic modulation, thermal modulation and magnetic modulation. However, the core of the THz modulator and dynamic devices lies in the semiconductor material, which determines the functionality and device performance. Different semiconductor materials can offer various functionalities in manipulating the spectral and spatial characteristics of the terahertz waves to form different types of modulator, such as amplitude modulators, phase modulators, polarization modulator, and programmable modulators. Therefore, we will focus on terahertz modulators based on the significant and representative semiconductor materials in this review.

**Figure 3:**
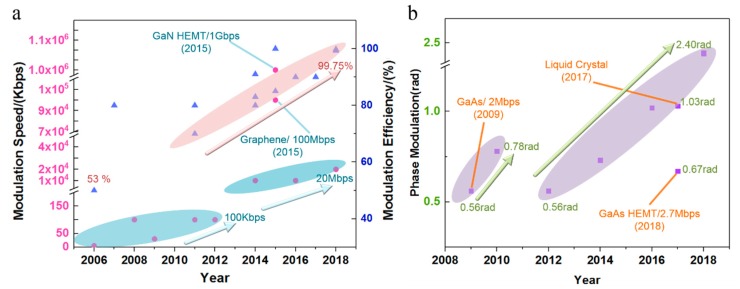
Progress of terahertz (THz) amplitude modulators (**a**) and transmitted phase modulators (**b**).

In general, the modulation speed and modulation depth are critical performance indicators for the modulators. Thus, many outstanding studies have been focused on improving the modulation speed, modulation depth and phase. More importantly, during the past 10 years, the performance of THz modulators has developed rapidly, and the modulation speed has increased from kHz to GHz, while the modulation depth increased from 40% to nearly 100% (Figure 3). The phase modulation ability of the transmission mode has been improved from 0.5 to 2.4 rad, while the reflection mode can reach to 2pi [7,8]. New kinds of modulators, such as on-chip modulators and compressing sensor modulators, have been proposed. Such achievements of THz modulators with active metasurfaces have already offered unprecedented functionality for manipulating THz waves.

Some of representative results of different kinds of THz modulators are shown in Table 1 and Table 2. Various materials have their own distinctive characteristics, opening new opportunities for terahertz manipulation. Such as the conventional bulk GaAs and silicon semiconductor modulator, it is easy to be manufactured and implement with external induced light or electrical field; the two-dimensional electron gas (2DEGs) and 2D material modulators have high speed and can be integrated; the liquid materials modulators have great potentials in the phase modulation and switches; the superconductor and phase transition materials modulators could realize intelligent temperature control switch.

Considering some outstanding previous reviews of terahertz dynamic metasurfaces which concentrated on functional classification [29,30,31,32,33] and different kinds of materials that have been included in the development of THz modulators, in this review article, we provide a brief overview of the various materials for the modulation of THz waves that have been investigated and demonstrated within the last few years.

The contents are organized as follows: Section 2 introduces THz wave modulation in conventional semiconductors and metasurfaces. In Section 3, we review the principle and application of 2DEG modulation and discuss recent developments in modulators based on two-dimensional materials in Section 4. Section 5 highlights recent research studies on VO_2_-based modulators before overviewing ferrite material systems and superconducting modulators in Section 6 and Section 7, respectively. The final section concludes with potential future developments.

This review might be useful for researchers who desire more knowledge regarding the developments and challenges of THz modulators and have devoted themselves to relevant research.

## 2. Conventional Bulk Semiconductor Metasurface

The earliest concepts of THz modulators based on active metasurfaces combine conventional bulk semiconductors and split ring resonator (SRR)-type metasurfaces. A bulk semiconductor can construct a Schottky diode structure with a metallic metasurface to act as the active component of this kind of THz modulator.

An external voltage is applied to the Schottky diode structure to tune the depletion characteristics of the Schottky junction while the depletion zone is modified. Therefore, the carrier concentration varies with a change in the external voltage, which leads to the modulation of dielectric properties so that the electro-magnetic resonant intensity of the metasurface can be modulated. With this mechanism, the amplitude value of the transmitted THz wave can be manipulated by electrically controlling this bulk semiconductor active metasurface.

Along with this concept, according to the Kramers–Kronig (K-K) relation, electrically controlled carrier concentration variations in such metasurface-semiconductor Schottky diode structures have been proposed to manipulate the phase of the transmitted THz waves. The change in the dielectric properties not only leads to amplitude modulation but also leads to phase modulation.

Moreover, in addition to the electrical diode structure, due to the photoconductivity of the semiconductor, a photo-induced THz modulator was proposed by applying an external laser beam. In this mechanism, the external laser will generate photo-induced carriers in the bulk semiconductors, which can be controlled by varying the incident laser power. By embedding photoconductive semiconductors within the gaps of the metasurface, the different carrier concentrations could lead to various resonant modes so that the transmitted and reflected THz waves can be manipulated. Similar to the electrically controlled modulator, the THz phase and amplitude modulator can also be developed by this mechanism.

### 2.1. Electrical Controlled THz Modulator

**Figure 4:**
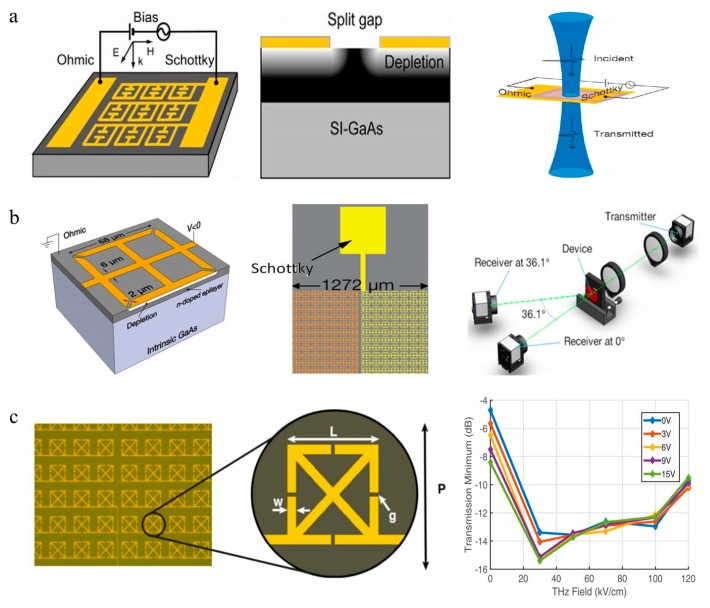
(**a**) Schematic of the THz metamaterial modulator. Reproduced with permission from [34], Copyright Springer Nature, 2006. (**b**) The diffractive modulator consists of 32 split ring resonator (SRRs) columns and a THz-TDS system is used to characterize the device. Reproduced with permission from [38], Copyright AIP Publishing, 2014. (**c**) Micrograph of the Metamaterial (MM) structure and incident THz field intensity influence transmission minimum at different applied DC biases. Reproduced with permission from [39], Copyright AIP Publishing, 2017.

The combination of metamaterials and doped semiconductors to achieve the amplitude modulation of THz waves first appeared in 2006 [34]. Chen et al. experimentally demonstrated an efficient THz modulator consisting of an array of SRRs patterned on a GaAs substrate (Figure 4a). The metamaterial structures were connected to serve as a Schottky gate; thus, the substrate charge carrier density could be real-time controlled by applying a voltage between the Schottky and ohmic contacts, thereby manipulating the THz waves. This device achieved 50% amplitude modulation at 0.72 THz with a reverse gate bias of 16 V. This work was the milestone step of THz modulator development, which opened the gate of combining the metasurface research area with the semiconductor materials research area. Based on the similar principle of the Schottky diode, the same team [9] reported a hybrid metamaterial phase modulator, obtaining a linear shift of 0.56 rad. Afterwards, researchers realized that the efficient combination of semiconductors and metasurfaces presents a reliable way to regulate terahertz waves [35,36,37,38,39]. D.M. Mittleman et al. experimentally demonstrated a THz diffractive modulator based on a planar metamaterial, which adopts a Schottky structure to adjust the resonance of structures. This device was composed of a diffraction grating and each column consisted of an SRR array, providing a dynamic range in excess of 20 dB through an applied alternate column voltage bias, as shown in Figure 4b [38]. In 2017, the same team designed an electrically modulated nonlinear metamaterial made of an SRR array grown on n-type gallium arsenide. The interaction between the electric field in the SRR and the carrier in the gallium arsenide cause a nonlinearity, which could be modulated by the voltage applied to the device (Figure 4c) [39]. In addition, a metamaterial modulator with two independent channels can actively modulate the terahertz waves of the corresponding channel by independently controlling the depletion zone of the two types of the Schottky structure under a bias voltage. The maximum modulation depth was ~46% and the modulation speed reached ~0.27 MHz [40].

### 2.2. Photo-Induced THz Modulator

**Figure 5:**
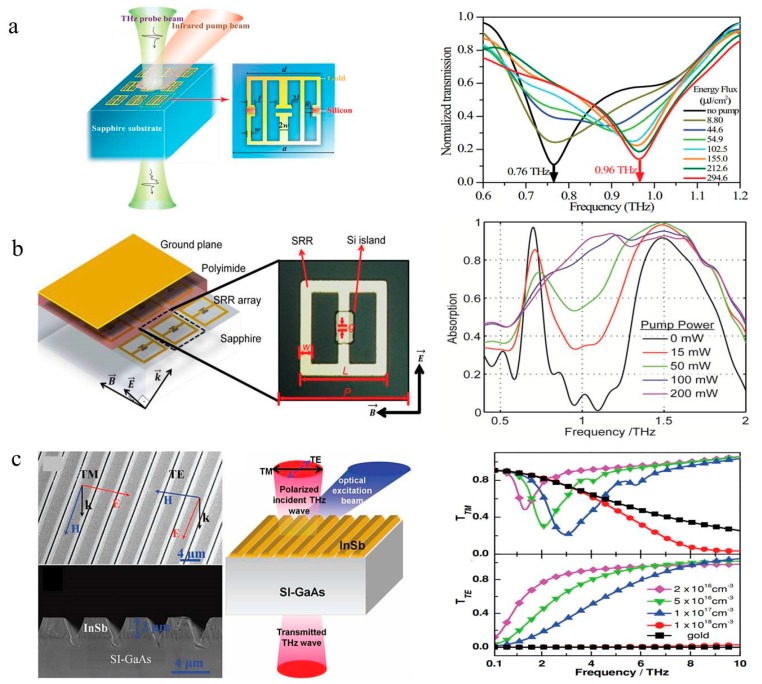
(**a**) Schematic of the metamaterial embedded with silicon in the gap. The resonant frequency shifts from 0.76 to 0.96 THz by changing the luminous flux. Reproduced with permission from [42], copyright American Physical Society, 2011. (**b**) Diagram of the absorber composition and results. The absorption intensity is tuned by applying different pump light intensity. Reproduced with permission from [43], copyright John Wiley and Sons, 2014. (**c**) Micrograph of the grating structure. with different electron concentrations, the transmission of TE and TM wave responses differently. Reproduced with permission from [44], copyright John Wiley and Sons, 2013.

As mentioned above, integrating a metamaterial and the photoconductivity of a semiconductor can realize an optically active metasurface terahertz modulator [41]. Shen et al. presented a broadband blueshift switch based on an electric-field-coupled inductor-capacitor (ELC) resonator, implying two potential resonance states (Figure 5a) [42]. The switch achieved a resonant frequency shift from 0.75 to 0.96 THz under an external laser beam, revealing potential applications of the device in THz systems. Similarly, a terahertz dynamic perfect absorber consisting of photoexcited carrier-changing silicon pads and split ring resonators was presented [43]. As shown in Figure 5b, by employing a ground plane to ensure the scarce transmission of the incident wave, a large reflecting modulation depth in two absorption bands is obtained. In addition to silicon, other materials such as InSb can also be utilized in terahertz modulators. A subwavelength InSb grating structure fabricated on a semi-insulating GaAs (SI-GaAs) substrate is presented (Figure 5c) [44]. Transverse Magnetic (TM) and Transverse Electric (TE) wave responses are shown when propagating through the grating. By changing the carrier concentration of InSb with laser pulses, the resonance frequency of the structure can be adjusted over a broad frequency range. This adjustment enabled the device to have a modulation depth of 46.7% and modulation speed up to potentially 1.2 GHz.

**Figure 6:**
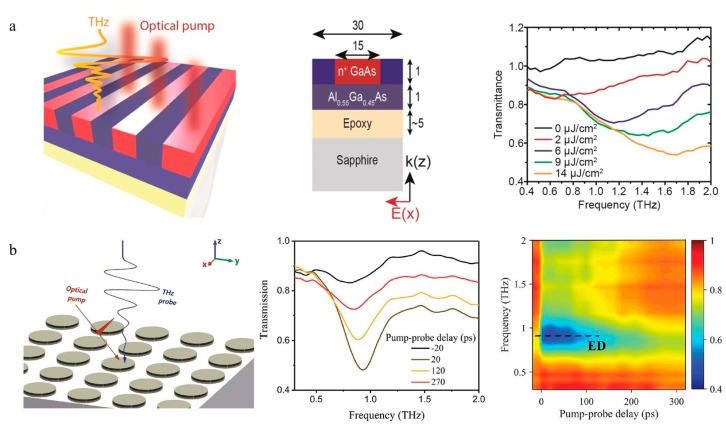
(**a**) Schematic of the optical-induced transient GaAs metasurface and the dimensions of the structure. The transmission coefficient of the THz wave declines with increasing pump fluence in the experiment. Reproduced with permission from [45], copyright American Chemical Society, 2017. (**b**) Structure of the tunable plasmonic metasurface. The figure on the right shows the experimental transmission spectra and transient transmission map of the plasmonic metasurface with different pump probe delay values. Reproduced with permission from [47], copyright John Wiley and Sons, 2018.

Recently, besides applying the control of photo-induced carriers in bulk semiconductors, light-matter interactions have also been used in THz modulators. To avoid inevitable absorption losses caused by metallic structures, the metasurfaces of the semiconductors have been heavily investigated. Noble semiconductors, such as GaAs [45,46], Si [47,48,49] and InAs [50], are the most common candidates among the dielectric metasurfaces. Yang et al. proposed a transient GaAs metasurface that can achieve a wide modulation band of the dipole resonance from 0.5 to 2 THz by controlling the carrier density of GaAs [45]. Although the 1D grating image is chosen to be the pattern, it should be emphasized that the plasmonic resonance of the metasurface is caused by the localized resonance rather than the grating effect, as shown in Figure 6a. Another transient plasmonic metasurface consisting of a Si disk array manufactured on a sapphire substrate was reported in 2018 [47]. The B^+^ ion implanted and annealed plasmonic metasurface provides a modulation depth up to 38% and ultrafast all-optical modulation of THz wave with a switch-on time of 20 ps (Figure 6b).

### 2.3. Coding Metasurface THz Modulator

**Figure 7:**
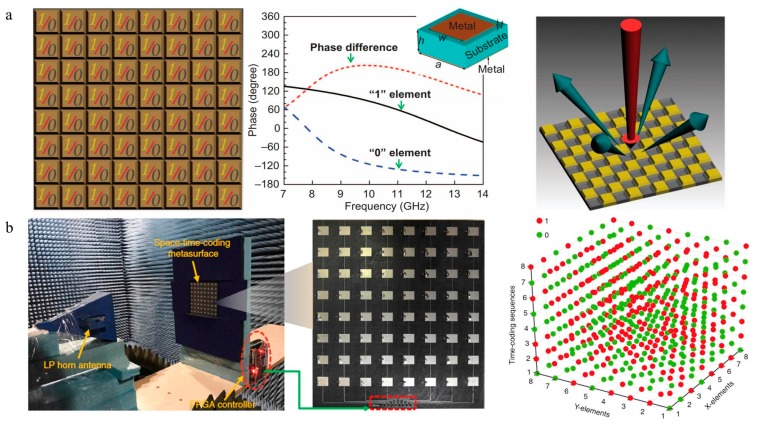
(**a**) A 1-bit digital coding metasurface. Reproduced with permission from [51], Springer Nature, 2018. (**b**) The space-time-coding digital metasurface and 3D coding matrix. Reproduced with permission from [63], copyright Springer Nature, 2018.

Digital coding and programmable metasurfaces based on PIN diodes have rapidly evolved since they were initially proposed in 2014 [51]. Cui et al. proposed a new concept of artificial “coding metamaterials,” which can be described, analyzed and designed in a digital way. The special metasurface particles are designed as ‘0’ and ‘1’ codes denoting opposite phases (Figure 7a). After integration with active elements (e.g., PIN diodes and Micro-Electro-Mechanical System(MEMS)), coding metasurfaces can generate different code sequences in real time under the control of a field-programmable (FPGA). Thus, compared with conventional metamaterials based on effective medium theory, the functionality of coding metamaterials can be controlled by binary code sequences, simplifying the design process and difficulty. Multi-bit coding metasurfaces based on the Minkowski closed-loop have been experimentally demonstrated to freely manipulate the scattering beams and the desired broadband diffusion of terahertz waves [52]. The theory and algorithm of information science have been directly applied to the description and design of coding metamaterials, which not only builds a bridge between the physical and digital worlds but also brings a series of novel discoveries and applications, such as reprogrammable holograms [53], vortex beams [54,55], reflection/transmission arrays [56,57,58,59,60], and diffuse scattering [52,61,62]. In 2018, combining the digital coding metasurfaces and time-modulated arrays, this team reported a space-time-coding digital metasurface to simultaneously control electromagnetic waves in both spatial and frequency domains [63]. By introducing time-dimension coding sequences, the number of conventional spatially coding can be extended, which reduces the complexity of designing multi-bit programmable metasurfaces (Figure 7b).

**Figure 8:**
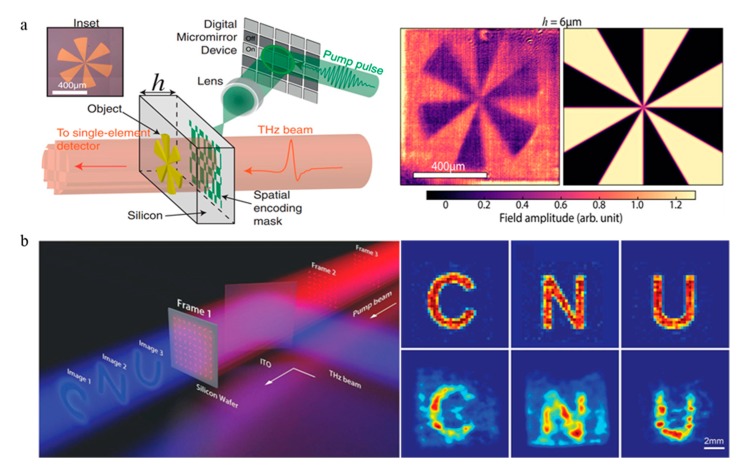
(**a**) Modulation imaging using digital micromirror equipment, which spatially modulates a coincident THz pulse. The terahertz waves of a sample are then measured by a single detector and the imaging result of a 6-μm-thick silicon wafer. Reproduced with permission from [67]. (**b**) Schematic representation of pure phase spatial THz modulator; and theoretical and experimental effects of dynamic holograms. Reproduced with permission from [72], copyright John Wiley and Sons, 2019.

Compressed sensing imaging based on programmable metasurface is an important development direction for terahertz imaging. Compressed sensing [64] completes the compression of information at the same time as information acquisition, breaking through the traditional Quinister sampling law, which can restore the total amount of information at a low sampling rate [65]. In 2014, Claire M. Watts published an article on the application of array-encoded modulators combined with compressed sensing to achieve 64-pixel image imaging [66]. More recently, another approach to form near-field THz imaging was proposed using a patterned optical pump beam, which induces the carrier distribution on the silicon wafer to selectively attenuate part of the incident light [67], which also achieves terahertz compression imaging through an encoded array (Figure 8a). In addition to amplitude modulation imaging, an important application of phase modulation is hologram [68]. In a conventional digital holography (CGH) design, the phase profile is controlled by etching different depths on a transparent substrate, but there is a problem that double image generation cannot be avoided. Metasurface provides an alternative to a simple and efficient hologram, initially applied in the microwave and visible light bands [69,70]. In 2017, Cui et al. proposed a coding metasurface-generated hologram to verify the feasibility of implementing multiple holograms with only one metasurface [53]. After that, the hologram of the terahertz band has also been greatly developed. A holographic metasurface that simultaneously regulates phase and amplitude was presented, realizing multiple longitudinal operations of holograms [71]. Recently, Chen et al. proposed a new method for generating wavefronts of arbitrary THz beams, as shown in Figure 8b. The hologram and zoom lens can be realized in real time by dynamically controlling the direction of the resonator [72]. Except as the dielectric metasurface provided, the metal metasurface also has outstanding performance in holographic imaging [73,74]. Since terahertz digital holography (THz-DH) has good resistance to light scattering and absorption, these results are expected to promote non-destructive testing of opaque soft materials.

## 3. Two-Dimensional Electron Gas Metasurface

In recent years, the amplitude and phase modulation have been realized by metamaterial devices that rely on conventional semiconductors, showing the application prospects in high-speed communication [75] and imaging systems [76]. To date, due to the mobility of the materials, the modulation speed of these terahertz modulators is in the MHz range, which limits the development of high-speed and low-voltage modulators. To develop high-speed dynamic terahertz modulators, researchers have focused on 2DEG with high mobility. 2DEG is usually induced by spontaneous polarization and piezoelectric polarization in the heterostructure [77]. Electrons confined to the modulation-doped heterostructure exhibit high mobilities since the 2DEG in the potential well is on the side of the intrinsic semiconductor, mitigating the deleterious effect of ionized impurity scattering. A high electron mobility transistor (HEMT) with excellent performance is a field effect transistor that utilizes 2DEG to work. The large-scale commercial application of HEMTs began in 1986 as a low-noise amplifier used in broadcast satellite receivers. This application laid the foundation for the development of microwave and millimeter-wave solid state devices, especially in the field of mobile communication and radar. These applications and studies promote the development of HEMTs and allow the realization of increasingly more advantages. In recent years, the carrier mobility of the 2DEG in HEMTs has reached more than 2500 cm/(V·s), while the carrier concentration is above 10^13^/cm^2^ and the operating voltage of the HEMT is usually several volts, which brings an excellent prospect for developing THz modulators. The 2DEG-based electronically dynamic terahertz modulator is used to set the HEMT to the critical position of each structural unit, therefore forming a block-shaped dynamic modulation region. There are thousands of periodic arrays of transistors and artificial microstructure arrays in the modulator chip. Through this ingenious combination, the artificial microstructure array acts as both a frequency selective surface and a transistor control circuit, thereby reducing the structural complexity and greatly reducing the insertion loss.

**Figure 9:**
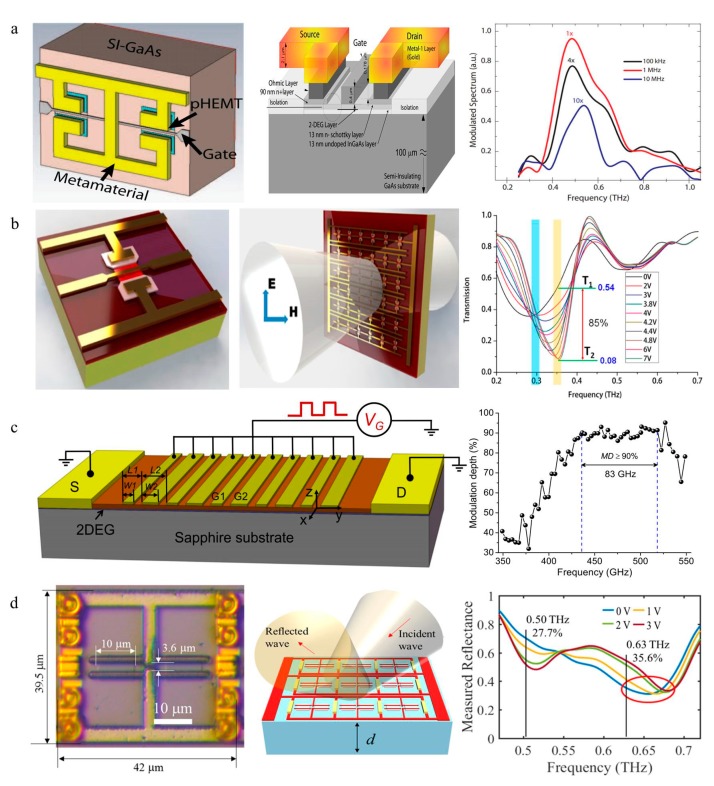
(**a**) A unit cell of the high electron mobility transistor (HEMT)/metamaterial device. The modulation speed of this device is up to 10 MHz. Reproduced with permission from [78]. (**b**) A unit cell of the modulator that utilizes voltage to convert different dipolar resonances. The transmission of the device at different voltages. Reproduced with permission from [14], copyright American Chemical Society, 2015. (**c**) Three-dimensional schematic view of the plasmonic terahertz modulator and modulation depth at different voltages [79], copyright AIP Publishing, 2016. (**d**) Schematic view of the modulator. The figure on the right shows the measured reflectance characteristics of this metasurface. Reproduced with permission from [80].

In 2011, Willie J. Padilla’s group from Boston University first demonstrated a combination of GaAs HEMTs and metamaterials to prepare terahertz dynamic devices, as shown in Figure 9a. In this work, the lnGaAs/GaAs HEMT is used as a key control region for the unit cell of metamaterials, and the 2DEG concentration in the channel is changed via the gate-voltage to control the metamaterial resonance. This modulator achieves a 33% modulation depth and operates at a high speed (~10 MHz) with a low operating voltage (1 V). This design concept promotes the development of terahertz modulators with respect to integration, low power consumption and high speed [78]. More importantly, this concept first brought the HEMT metasurface to THz development.

Following this concept, considering the wide band gap, high electron mobility and saturation velocity of GaN in comparison to the 2nd-generation semiconductor GaAs, the GaN-HEMT makes it an ideal candidate for high-performance THz dynamic devices. In 2015, Zhang et al. presented a composite metamaterial structure based on an InAlN/AlN/GaN/AlN/GaN double-channel heterostructure to obtain an ultrafast THz modulator [14]. This work greatly improved the modulation speed and depth by using voltage to convert different dipolar resonances. Not only was a phase shift of 1.19 rad be realized, but more importantly, a modulation speed of 1 GHz and a modulation depth of 85% were achieved for the first time in the real-time dynamic test (Figure 9b). This was the first time that the modulation speed of the THz modulator reached 1 GHz. Subsequently, Zhang X. et al. proposed a single channel AlGaN/GaN HEMT-metasurface THz modulator. This device achieved a 33% modulation depth and a 20 MHz modulation speed at a higher operating frequency of 0.835 THz [81].

In addition, the capacitive properties of HEMTs are also used for manipulating THz waves. Nouman et al. fabricated an AlGaAs/InGaAs heterostructure to act as a metal semiconductor metal (MSM) 2DEG-varactor located at the center of the SRR structure. By varying the applied voltage (0~3 V) to the MSM 2DEG-varactor, the resonance frequency of the SRR-based metamaterial was altered from 0.52 to 0.56 THz. This device obtained a 13% modulation depth with an insertion loss of 4.3 dB at 0.58 THz, and the theoretical 3 dB cut-off frequency was 48 MHz by calculating the (Resistance-Capacitance) RC constant (3.2 ns) [82]. On this basis, the team combined a metasurface with the Fabry–Perot cavity to obtain a reflection mode THz modulator to increase the modulation depth. Benefiting from the resonance enhancement effect of the Fabry–Perot cavity, the modulation depth at 0.58 THz increased to 30% (Figure 9d) [80].

Huang Y. D. et al. placed a metal deep subwavelength periodic grating gate on the GaN/AlGaN 2DEG channel. As shown in Figure 9c, under a gate bias, the equilibrium electron density can be periodically modulated, resulting in tunable 2D plasmonic cavities underneath the grating gate. By manipulating the interaction between terahertz EM waves and 2D plasmons, this collective electron plasma excitation THz modulator achieved a modulation depth of at least 90% over a spectrum bandwidth of 83 GHz (435.6~518.4 GHz) and a 400 kHz 3 dB operation bandwidth [79].

Based on these studies, the active HEMT-metasurface elevated THz modulators to a higher step compared with conventional bulk semiconductors. In addition, phase control is important for the phase shift keying modulation of THz wireless communication and THz imaging systems, while high speed and phase tunable terahertz modulators are essential and urgent. On the basis of these investigations, THz phase modulators based on an active HEMT-metasurface have been proposed in recent years. Various innovative metamaterial structures combined with 2DEG heterostructures have been developed to increase the THz phase shift.

In 2017, Zhou et al. implanted a delta-doped double pseudomorphic heterostructure into a kind of symmetric quadruple-SRRs metamaterial structure to fabricate an electrically controlled THz modulator [15]. Due to the symmetry of the metamaterial element, all magnetic responses were cancelled; furthermore, a strong purely electric response was revealed by incident THz waves. By tuning the conductivity of the HEMT, the LC resonance strength in the metamaterial could be controlled to modulate the THz wave. A modulation speed of 2.7 MHz with 80% modulation depth at 0.86 THz and a phase shift of 0.67 rad at 0.77 THz were realized under a reverse voltage of −4 V.

**Figure 10:**
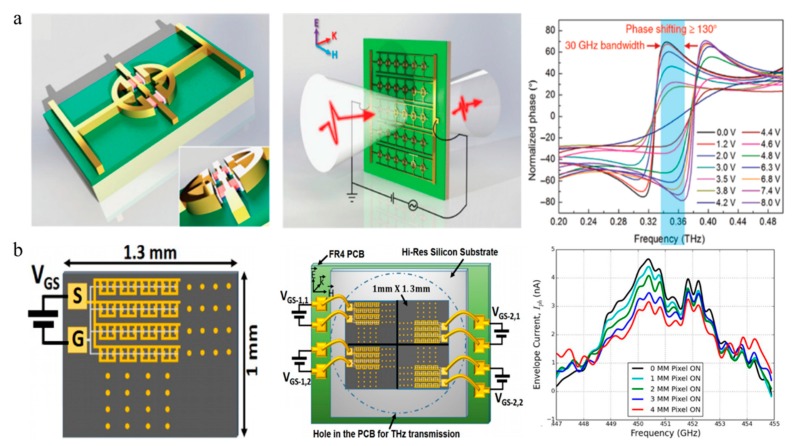
(**a**) Unit structure of THz phase modulator. Phase of incident THz wave can shift by applying different voltages. Reproduced with permission from [25]. (**b**) Structure of the terahertz wave-front modulator. Characterization when sequentially turning “on” each pixel of the spatial light modulator (SLM). Reproduced with permission from [83].

In 2018, Zhang et al. performed large phase modulation by enhancing the resonance of an active HEMT metasurface [25], as shown in Figure 10a. According to the K-K relation, the relationship between the resonance intensity change and the phase jump change was investigated, and the results indicate that the stronger resonant intensity corresponds to the larger phase jump [9]. By comparing various resonant structures, the enhanced inductance-capacitance dipole resonance (LCDR) resonant structure was embedded with a 2DEG layer of GaN HEMT. Thus, the carrier distribution and density of the 2DEG can be tuned to dynamically manipulate the resonance intensity and surface current circuit of the resonance mode, leading to a 137° phase shift and a 2.4 GHz modulation speed in the dynamic experiments.

Based on the previous active terahertz metamaterials, S. Rout reported a transmissive terahertz spatial light modulator (SLM) consisting of a 2 × 2 pixel array [83] (Figure 10b). Single pixel imaging experiments were performed, demonstrating spatial modulation with low voltage (1 V) and low power (<1 mW). However, the design of the modulator circuit cannot achieve high pixel and large array imaging, while crosstalk still exists between multiple pixels.

## 4. Graphene and 2D Material Metasurfaces

Graphene was the first successfully isolated and atomically thin 2D material, opening the door to the world of 2D materials [84]. Due to its linear wave-vector relationship with zero bandgap, graphene has unique optical and optoelectronic properties, such as tunable carrier densities and negative dynamic conductivity under optical pumping [85,86,87]. Furthermore, the mobility in graphene film can be as high as 10^5^ cm^2^/Vs at room temperature [88] with carrier concentration modulations up to 10^14^ cm^-2^ [89]. These properties make graphene attr active because of their potential in the development of high-speed electronic devices. The optical conductivity of graphene considering only the intra-band contribution at THz frequency is closely related to the Fermi energy. In practical applications, varying the Fermi energy of graphene causes the carrier concentration to change, which means that the conductivity can be controlled to modulate the transmission characteristics of terahertz waves.

**Figure 11:**
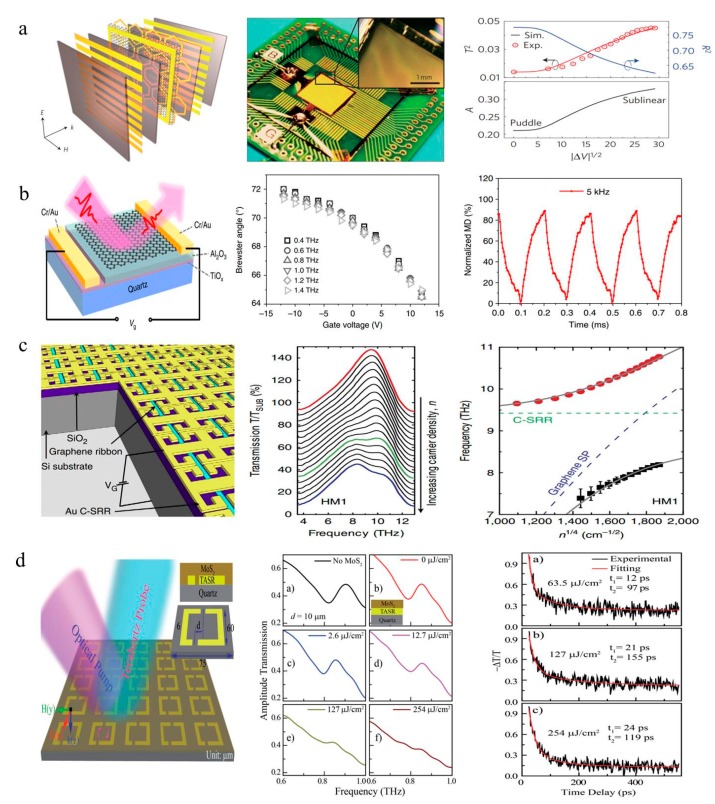
(**a**) Schematic view and device images of the active graphene modulator. Reproduced with permission from [23], copyright Springer Nature, 2012. (**b**) The Brewster angle as a function of gate voltage and modulation speed of the modulator. Reproduced with permission from [94], copyright Springer Nature, 2018. (**c**) Transmission modulator of C-SRR-GR hybrid metamaterials. Reproduced with permission from [99]. (**d**) Transmission spectra of the device and the transient dynamics of MoS_2_ for different pump intensity. Reproduced with permission from [13], copyright John Wiley and Sons, 2017.

Generally, graphene modulators are classified by electrically driven or optically driven modulators. In 2012, Sensale-Rodriguez et al. reported some significant studies on graphene THz modulators igniting the field [90,91]. At the same time, an integrated device based on gate-controlled active graphene metamaterials was demonstrated [23]. This device was composed of a single layer of graphene fabricated on a hexagonal metallic frame and top/bottom thin metallic wire array electrodes embedded in a dielectric material, as shown in Figure 11a. The measured modulation amplitude and phase of the transmitted wave reached approximately 47% and 32.2°, respectively. Although this work was the first to propose a compact modulator that can be implemented on an integrated printed circuit board, its driving voltage is as high as ~350 V, which severely limits any possible application of this device. To address this issue and improve the modulation depth, various groups have conducted a series of studies and have achieved outstanding results [92,93,94,95,96]. In a very recent study, Chen et al. demonstrated that the Brewster angle of a graphene/Al_2_O_3_/TiO*_x_* sandwich structure can be tuned by varying the conductivity of the graphene, as shown in Figure 11b. In this way, an ultra-broadband THz intensity modulation with amplitude modulation larger than 99.3% and a phase tunability up to 140° from 0.5 to 1.6 THz was achieved [94]. Arrays of graphene modulators can be employed for THz imaging applications [97]. Graphene is also a favorable material in plasmonic structures for manipulating terahertz waves due to its primitive frequency response [98]. Hybrid metamaterial structures comprising graphene resonators and metallic SRRs are employed as high-speed THz modulators, exhibiting 60% modulation of the peak transmission (Figure 11c) [99]. The graphene-localized SP resonance is tuned to achieve strong near-field coupling with a C-SRR LC-resonance by electrically varying the carrier density. Metamaterial structures with graphene [100,101,102,103], stacked multilayer structures [104], plasmonic waveguide structures [105,106] and other hybrid structures [107,108,109] have attracted considerable attention for light modulation and improved modulation performance.

In addition, all-optical graphene modulators have been extensively studied as an important supplementary modulation method because they enable direct modulation in optical fibers or waveguide systems. Here, some all-optical active modulators including graphene-clad microfibers [110], all-fiber modulators with Mach–Zehnder interferometer structures [111] and other structures [112], were mainly demonstrated.

### Other 2D Materials

Although the existence of a Dirac point gives graphene a number of interesting properties, it also hampers its application in the semiconductor field. The FETs made of graphene are not well used in practice with their small switching ratio. To further explore the world of 2D materials, a variety of 2D materials have been successively isolated, including hexagonal boron nitride (h-BN) with a wide bandgap [113], transition metal dichalcogenides (TMDs) with a direct bandgap [114,115], and black phosphorus [116]. There are similarities and differences in the properties between graphene and other materials. For instance, similar to graphene, monolayer TMDs have mechanical flexibility, thermal stability and high electron mobility. However, owing to the band gap resonance, the optical absorption in 2D TMDs is stronger than that in graphene, reaching up to 10%.

In principle, other 2D materials beyond graphene can also be effective for the active modulation of terahertz waves. As a typical TMD, molybdenum disulfide (MoS_2_) has already been heavily reported for its unique properties in THz applications [117,118]. Cao et al. reported a terahertz modulator based on multilayer MoS_2_ and silicon with higher modulation performance than a graphene-based modulator [119]. The optical modulator had a modulation depth of 96% under a pump power of 4.56 W. In 2017, Srivastava Y K et al. demonstrated that the ultrasensitive active switching and modulation of Fano resonances can be realized by integrating MoS_2_ with metamaterial [13]. The Fano resonance amplitude gradually decreased with increasing optical pump power and eventually disappeared, achieving a 100% modulation depth at a pump power of 200 mW. Notably, the drop-casted MoS_2_ active metamaterial device can switch the Fano resonance on the time scale of 100 ps, as shown in Figure 11d.

## 5. Vanadium Dioxide Metasurface

Among the THz modulators, a vanadium dioxide (VO_2_) metasurface is one of the hottest topics. VO_2_ exhibits an insulator-to-metal transition upon heating, which was reported by Morin, F.J. in 1959 [120]. This phenomenon is due to the atomic rearrangement of VO_2_, which transforms from the low-T monoclinic phase to the high-T rutile phase when the temperature increases. Then, experiments showed that this transition can also be triggered by femtosecond light pulses [121,122] and an electric field [123]. Compared with transistors and 2D materials, the ease of fabrication and sub-picosecond response time of VO_2_ provide a promising approach for tunable devices in the terahertz range. By applying the phase transition characteristics, T. Driscoll’s group first presented the THz memory metamaterial based on the VO_2_ metasurface in 2009 [124]. Later, ultra-strong THz pulses were proposed to induce the phase transition of the VO_2_ metasurface within an ordinary SRR structure [125]. Such advanced work made us realize that VO_2_ metasurfaces can be utilized to reconfigure the meta-unit structure and achieve mode conversion at the surface so that we can manipulate the THz waves. Therefore, by applying the phase transition characteristics, an increasing number of THz dynamic devices have been proposed. Research on THz modulators based on VO_2_ has mainly focused on amplitude modulation and phase modulation.

Using an early design, pure VO_2_ film, which was shaped into cut-wire structures fabricated on a semiconductor substrate, construct a simple but practical THz amplitude modulator [126]. When an external laser or temperature source is not loaded, the VO_2_ film acts as a transparent material to the incident THz wave, and the loss is approximately –1.4–1.8 dB. When the phase transition is triggered, the VO_2_ film transfers into the metallic state so that the THz wave cannot transmit through the film. This approach is a very practical way to realize the modulation of THz waves. More importantly, the modulation operating band is sufficiently wide.

**Figure 12:**
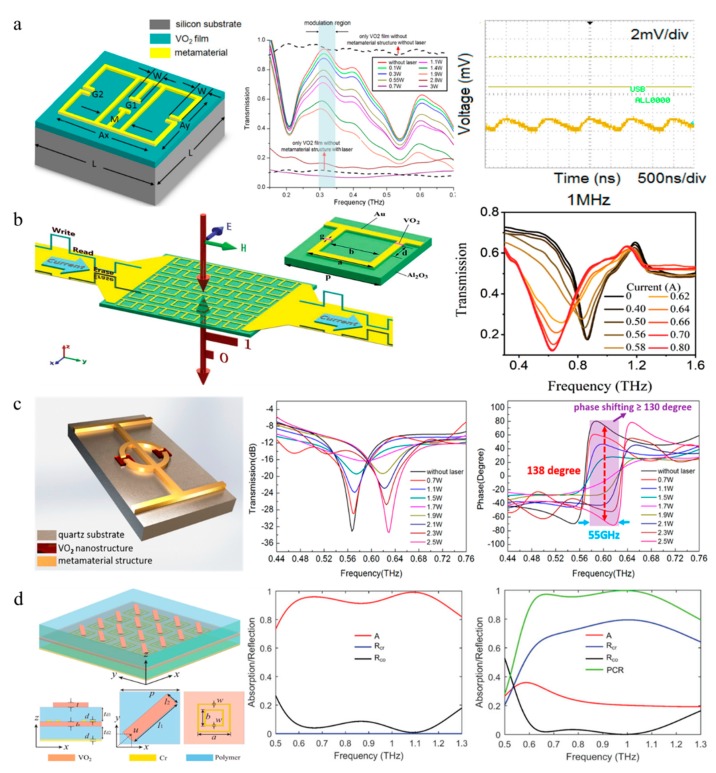
(**a**) Schematics for one unit. The transmission of the device decreases with increasing laser-power. The planes on the right shows the output of a 1 MHz pump laser signal. Reproduced with permission from [130]. (**b**) Schematic of the multifunctional metasurface and experimental transmission with different applied currents. Reproduced with permission from [132], copyright John Wiley and Sons, 2018. (**c**) Three dimensions of one cell and experimental results of the transmission spectra and phase spectra with TDS. Reproduced with permission from [27], copyright, American Chemical Society, 2018. (**d**) Structure of the multifunctional metasurface and the simulated data of vanadium dioxide (VO_2_) in an insulating state and a fully metallic state are also illustrated. Reproduced with permission from [140], copyright John Wiley and Sons, 2018.

Hybrid metamaterial terahertz devices, which combine the static frequency response of the metamaterial to the electromagnetic wave with the phase transition of VO_2_, have realized the real-time dynamic modulation of THz waves via various excitations [127,128,129]. For instance, a THz amplitude modulator combining a VO_2_ film and a dual-resonance metamaterial was proposed [16]. The phase transition under illumination of the VO_2_ films between the metallic structure and substrate results in a change of the transmittance of the THz wave. At the same time, the symmetric dual-resonance units allow high transmission over the designed ultra-wide band during a static experiment (Figure 12a) [16]. However, the modulation speed is limited by the phase change recovery rate of the VO_2_ in the dynamic test. More recent work has demonstrated that the electrical bias tuning effect is attributed to ohmic heating [130]. A multifunctional integrated device is very desirable for optical devices in any frequency band. Mayer et al. demonstrated a hybrid metamaterial platform that achieved electrically switchable reflection, pixelated light manipulation and memory effect control in the mid-infrared region [131]. In the terahertz range, a multifunctional meta-device based on VO_2_ has also been proposed recently (Figure 12b) [132]. The dynamic device exhibits ultrafast switching excited by a femtosecond pulse, which is much faster than the electrical type in the second level. Furthermore, due to the hysteresis characteristic of VO_2_, the hybrid metasurface plays another important role of the THz memory device. More recently, a square-loop metamaterial based on W-doped VO_2_ was reported, which realized transmission modulation with a lower transition temperature than that of pure VO_2_, while the modulation depth was lower as well [133]. In addition to the metasurface, a photonic crystal waveguide coated by a VO_2_ film [134] and an undulated waveguide integrated with a VO_2_ film [135] have also been reported.

THz phase modulation is an attractive but difficult research direction for terahertz modulators, aiming to achieve large and continuous phase modulation with low loss. In 2016, according to Babinet’s principle, a reconfigurable metamaterial converts the metal structure to the corresponding complementary structure through the VO_2_ phase transition, obtaining a π/2 phase shifter for terahertz waves in the same polarization direction [136]. In 2018, by optimizing the design of artificial microstructures and analyzing various coupling resonant modes, Zhang et al. proposed a ring-dumbbell hybrid meta-nanostructure combined with VO_2_ nanostructures (Figure 12c) to realize phase shifting [27]. With different laser powers, the phase transition of VO_2_ changes the resonant mode of the metasurface, leading to a remarkable phase shift up to 138° at 0.6 THz. An average phase shift of 130° over 55 GHz is a considerable improvement that has not been previously reported, but the high loss is also a problem that needs attention. Another electronically controlled phase shifter with a similar structure was also reported in the same year [137].

Metasurfaces integrated with VO_2_ not only enable amplitude and phase modulation of terahertz waves, but also realize other functionalities, such as a THz quarter-wave plate and chirality manipulation [138,139]. In another example, the switchable metasurface is composed of different functional layers with diversified functionalities, which can realize a wide-band absorber or a reflective wide-band half-wave plate by utilizing the phase transition of VO_2_ [140]. The absorption of this device exceeded 90% in the range of 0.562 to 1.232 THz, as shown in Figure 12d, or it can obtain a high conversion efficiency of the linear polarization wave with over a 60% reflectance at a 0.49 THz-width band while VO_2_ turns into metallic-state.

## 6. Liquid Crystal Metasurface

**Figure 13:**
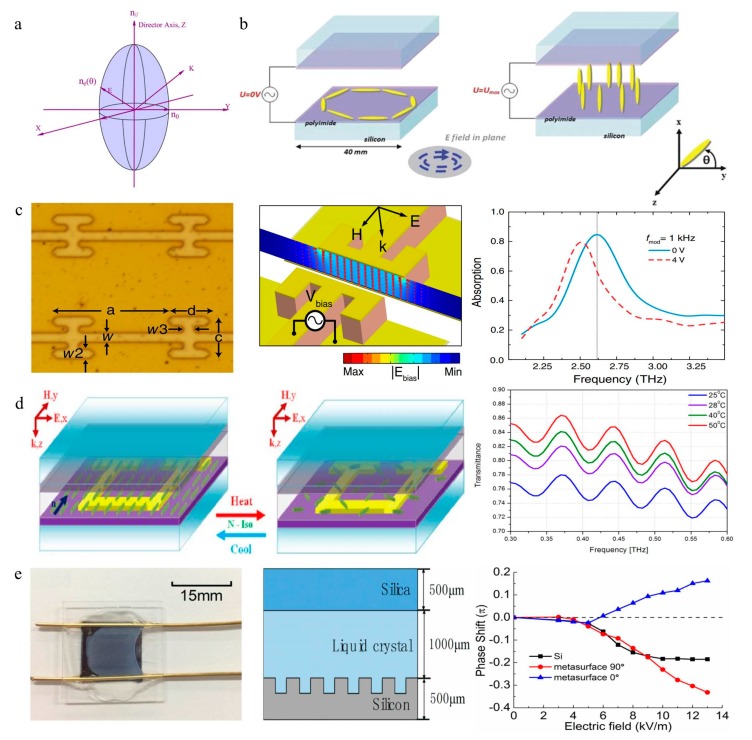
(**a**) Schematic diagram of the interaction between polarized light and liquid crystal molecules. Reproduced with permission from [153], copyright Elsevier, 2009. (**b**) Structure of the electrical adjustability of liquid crystal molecules. Reproduced with permission from [143]. (**c**) The device consisting of an electrically controlled liquid crystal and metamaterial absorber structure provides an absorption tuning of 30% at 2.62 THz. Reproduced with permission from [142], copyright American Physical Society, 2013. (**d**) Schematic diagram and resulting temperature control structure combining the liquid crystal and metamaterial transmission modulator. Reproduced with permission from [151]. (**e**) Electrically controlled phase shifter composed of the liquid crystal and metasurface can obtain phase shifting with varying electric field power. Reproduced with permission from [28].

Both fluidity and molecular order co-exist in a liquid crystal [141]. Thus, the director distribution and optical properties of liquid crystals strongly depend on the surface effect and ambient temperature, and its dielectric anisotropy covers a wide frequency range. Additionally, as a tunable electro-optic material, it is dielectric anisotropic from ultraviolet to microwave, which renders it an excellent tunable electro-optic material. The liquid crystal has a birefringence effect [142]. When natural light is incident on a liquid crystal, it will be decomposed into two kinds of polarized light, whose vibrating surfaces are perpendicular to each other. The one whose vibration direction is perpendicular to the optical axis is called ordinary light, and its refractive index is n_0_. The vibration direction of the other kind of light, which is called extraordinary light, is parallel to the optical axis, and the extraordinary light refractive index is n_e_ (Figure 13a). The large birefringence characteristic (∆n = n_e_ − n_0_) makes the liquid crystal sensitive to the polarization of light. Furthermore, these materials have good electrical controllability in the terahertz frequency band [143]. The liquid crystal molecular array tends to the lowest potential state and can orient a molecular arrangement to the applied electric field, pointing in the same direction as the electric field line (Figure 13b). Due to the dielectric and optical anisotropy, liquid crystal materials have great application potential. The direction of liquid crystal molecules can be adjusted through an external field to effectively regulate the intensity, phase, and polarization of electromagnetic waves in various frequency bands [18,28,144,145,146,147,148]. In the terahertz range, although the large absorption loss and scattering are problems, the birefringence characteristics of liquid crystals is much larger than that of ordinary anisotropic materials. Therefore, liquid crystals have a quite high research value in the terahertz bands.

The birefringence characteristics of liquid crystals and the remarkable electro-optic tunability have been applied to terahertz modulators. In recent years, liquid crystal-based terahertz electronically controlled absorbers have been developed. In 2016, David Shrekenhamer added the liquid crystal into the metamaterial unit, achieving a wide range of absorption tuning and a certain resonance broadband absorption tuning [142], indicating that the all-electronic method can dynamically control the basic light interaction with the surface (Figure 13c). In addition, there are some similar metamaterial structures combined with liquid crystals, such as complementary SRRs [146], hybrid re-configurable 3D structures [147] and cross-shaped metamaterials [18]. Although a high modulation depth can be obtained through the combination, the response time of a conventional liquid crystal is long. To make full use of the high birefringence of a liquid crystal and improve the liquid crystal response time, Yin introduced a dynamic metamaterial absorber with a polymer network liquid crystal (PNLC) in 2018 [149]. The peak resonant frequency of the absorption spectra shows a shift by electrically controlling the direction of the PNLC embedded in the metamaterial. Furthermore, the adjustment time (10 ms) and recovery time (85 ms) of the PNLC-based metamaterial absorber are significantly faster than those of traditional nematic liquid crystal tunable metamaterial devices. In addition to the metal metasurface, a liquid crystal can also be combined with the metasurface of the medium. Zhou proposed an absorber structure in which graphene was used as an electrode that sandwiched a liquid crystal embedded in a silicon column [150]. By changing the bias voltage, the liquid crystal orientation could be adjusted continuously. Under the condition that the bias was saturated, the liquid crystal was driven vertically, reaching an absorption peak of 0.86 at 0.79 THz with a modulation depth of 47%.

The refractive index of liquid crystals is also temperature adjustable. In 2018, Kowerdziej demonstrated the tunability of thermally induced liquid crystal metamaterials [151], as shown in Figure 13d. The thermal tunability of this metamaterial device is attributed to the temperature sensitivity of the liquid crystal dielectric constant contained in the metamaterial cavity. The experimental results showed that the resonant response of a metamaterial device can be effectively tuned with respect to its size and wavelength, and its spectral tunability is close to the theoretical limit of 8 GHz. The development of single-function liquid crystal metamaterials has also promoted the emergence of multifunctional liquid crystal metamaterials. Shen proposed an integrated device for the EIT device in the transmission mode and the absorber in reflective mode. The liquid crystal was used as an intermediate medium with an adjustable refractive index. Through a variation in the voltage, the liquid crystal could be redirected to achieve fast active tuning, whose modulation depth is 37% at 1.27 THz, and the tune of absorption was 15% at 1.30 THz [152].

Liquid crystals can be applied to phase shifters by controlling the switching between n_0_ and n_e_ with voltage. Altmann demonstrated that a phase shift could be achieved over 2.5 THz with a polymer-stabilized liquid crystal of 95% liquid crystal and 5% polymer and a reduced response time [144]. A phase shift of 360° was achieved at 684 GHz when the threshold voltage ranged from 5 to 45 V. In previous studies of liquid crystal phase shifters, the alignment processing of liquid crystal molecules is required to reduce losses. Tomoyuki proved that a graphene electrode and a randomly arranged liquid crystal cell are suitable for voltage-controlled phase shifters, obtaining a phase shift of 0.11 rad at 1.5 THz [145]. In the terahertz band, the phase modulation depth of a liquid crystal under an external voltage is still limited, which causes the response to be slow, so a high voltage is needed. Thus, the combination of a liquid crystal and a metamaterial will be one of the research directions of new tunable terahertz devices. Yun realized a large artificial birefringence effect by combining a metasurface and a liquid crystal and obtained a phase shift of 0.33π at the bias voltage [28]. Compared with silicon without a metasurface, the artificial structure can enhance the liquid crystal phase shift in the terahertz band, as shown in Figure 13e.

## 7. Superconducting Metasurface

The ohmic loss of a metasurface is a nonnegligible problem as the frequency is pushed towards the terahertz region. Considering the unique advantages that a superconducting material brings to a metasurface, such as loss reduction, a higher figure of merit (FOM), and new phenomena of switching or modulation, superconducting materials have been widely used in the field of THz [154,155,156]. Furthermore, once the superconductor is in the superconducting state, it is highly sensitive to external excitation and easily tuned by illumination [157], electric current [158], magnetic field [159] and temperature [160], indicating a new opportunity for application in terahertz functional devices. Typical features of superconductors are the disappearance of the DC resistance and perfect diamagnetism below the critical temperature (Tc). According to the transition temperature, superconductors are classified into high-Tc superconductors (HTS), which are usually made from yttrium-barium-copper oxide (YBCO) films [161,162,163] and low-Tc superconductors, represented by niobium nitride (NbN) and niobium (Nb) [159,164,165,166,167]. Superconductors can also be introduced to superconducting metamaterials with a negative refractive index instead of metallic materials [168].

### 7.1. High-Tc Superconductors with a Dynamic Tunable Metasurface

**Figure 14:**
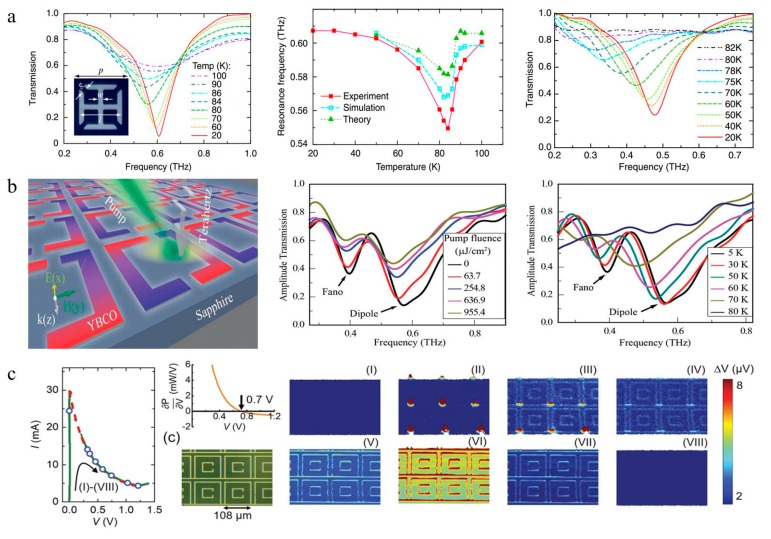
(**a**) Transmission amplitude spectrum at different temperatures, when the thickness of the yttrium-barium-copper oxide (YBCO) film is 180 and 50 nm [161], copyright American Physical Society, 2010. (**b**) An optical pump ultra-fast switch with YBCO based on a terahertz asymmetric Fano resonant ring [19], copyright John Wiley and Sons, 2018. (**c**) The measured I−V curve at 4.9 K and the low temperature scanning laser microscope (LTSLM) scan images at different DC bias voltages [164].

As a typical high-temperature superconducting material, YBCO was the first material discovered to become superconducting above 77 K, which lowered the cost of cooling the material below the critical temperature. However, the surface impedance of YBCO increases faster with frequency compared with metal, which is higher than that of Au above 0.5 THz at 4.2 K [169,170]. Thus, terahertz metamaterial devices based on YBCO can realize the manipulation of terahertz waves. Terahertz superconductor metamaterial devices consisting of SRR arrays have been demonstrated to modulate the transmission of waves, achieving resonance switching effects and frequency tuning by varying the temperature [155]. In 2010, H. T. Chen et al. of the Alamos Laboratory in the United States achieved a 35% amplitude modulation of the terahertz wave by etching the YBCO material into an artificial microstructure resonant ring, and they also found that the resonant frequency shifted when the thickness of the YBCO film changed (Figure 14a) [161]. In 2016, Keiser etched YBCO thin films into SRR arrays and designed a terahertz saturated absorber [171]. Under low electric fields, the adsorption reached 80% at a temperature of 10 K, and decreased to 20% at a temperature of 70 K. Impedance matching and the peak absorption of the absorber are reduced by changing the temperature or field intensity, which changes the complex conductivity of the SRR arrays. In 2018, considering that the Cooper pairs of YBCO dissociate and recombine in an extremely short time under irradiation, Ranjan et al. designed a two-channel ultrafast photonic switch using a terahertz asymmetric split ring metamaterial (Figure 14b). Their team conducted an all-optical modulation experiment using a two-channel and ultrafast device and demonstrated good performance. In terahertz high-speed wireless communication, dual-channel switchable devices have broad application prospects [19]. In the same year, Jing et al. used a low temperature scanning laser microscope (LTSLM) to image the transition from a superconducting to a normal state of a superconducting terahertz modulator by applying different bias voltages, proving that the thermal effect plays an important role in THz transmission modulation (Figure 14c). This characteristic has some reference value for improving the modulation speed [164].

### 7.2. Low-Tc Superconductors with a Dynamic Tunable Metasurface

Nb and NbN are typical low-temperature superconducting materials. Nb has extremely low surface resistance in the superconducting state, and its energy gap frequency is approximately 0.7 THz. NbN has a relatively high Tc and gap frequency. The gap frequency of NbN thin films is approximately 1.2 THz, much higher than that of Nb, which indicates that NbN can maintain low loss characteristics at higher frequencies [170].

**Figure 15:**
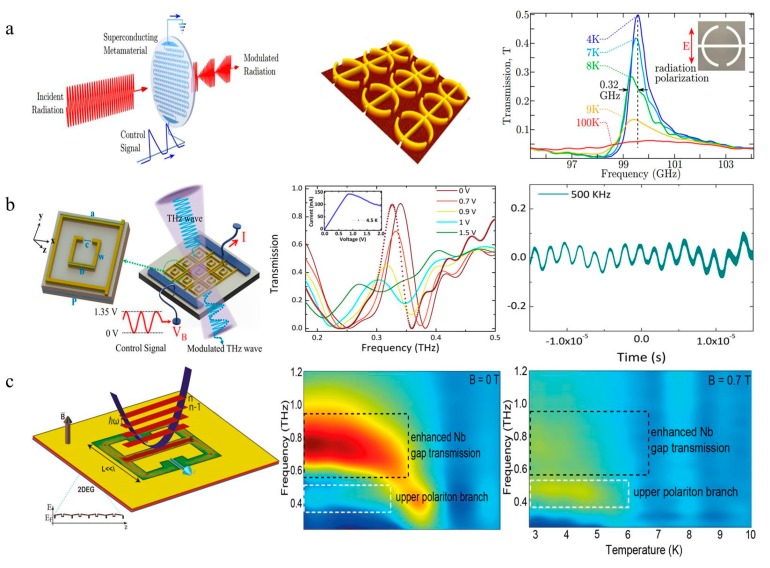
(**a**) A superconducting terahertz electrical modulator with a 100 kHz modulation rate based on the Fano resonance [172], copyright Physical Review Letters, 2012. (**b**) A superconducting terahertz modulator based on the electromagnetic induction transparent metamaterials [20]. (**c**) The light-matter coupling experimental device. The transmission of the switchable THz superconducting metasurface on a 2DEG changes by varying frequency and temperature [173], copyright Springer Nature, 2017.

In 2012, V. Savinov first designed a terahertz electrical modulator with a 100 kHz modulation rate and a 45% modulation depth based on a high-Q Fano resonance using a Nb film (Figure 15a) [172]. The magnetic field generated by controlling the current suppresses the superconductivity of the Nb. In a weak magnetic field, the transmission modulation is proportional to the amplitude of the control current, while the relationship between them is quadratic at a low modulation frequency with a thermal effect. Compared to Nb, NbN is a more suitable terahertz superconducting material with a higher gap frequency and wider tuning property [166]. In 2017, Chun Li and Biaobing Jin et al. proposed a switchable superconducting NbN metamaterial device with high switchable ratios (Figure 15b). Due to the quench property and the heat dissipation of the device, the modulation speed is 1 MHz which is the highest speed of the superconductive metasurface THz modulator [20]. There has been unique research on superconductors. Keller et al. found that superconducting properties may be altered by the presence of a two-dimensional electron gas (2-DEG). The system design is composed of a switchable THz superconducting metasurface forming the cavity which can be seen as an LC-circuit and can achieve high Q factors (Q = 54), and a two-dimensional electron gas (2-DEG) as the matter (Figure 15c) [173].

Based on the pioneering studies mentioned above, more novel modulation effects of superconducting metamaterials have been proposed to develop superconducting terahertz modulators, such as a nonlinear response [159,167,174,175] and superconducting plasma photonics and superconducting metamaterials with quantum effects [176,177,178].

## 8. Spintronics Metasurface

With the development of spintronics, a branch called THz spintronics has been emerging. Antiferromagnets have been intensively studied for a resonance frequency in the THz band. Thus, this kind of material has been proposed to be utilized in exploiting new types of THz modulators.

Antiferromagnets exhibit ultra-fast dynamic properties [179] in the terahertz range, which can produce a spin effect to tune terahertz waves by external factors and realize the switching of modes and the spin precession of the antiferromagnet [180]. Based on this feature, antiferromagnets are widely used in the research of spin reorientation, coherent control and nonlinear dynamics combined with metamaterials. In the tilting antiferromagnetic structure of RFeO_3_ (R = Y, Nd, Dy, etc.), the quasi-ferromagnetic mode (F mode) and quasi-antiferromagnetic mode (AF mode) are excited by the magnetic field of the THz pulse. THz-TDS can detect the spin reorientation process of the macroscopic magnetization direction under external triggering [181,182].

Nakajima et al. studied the coherent control of ferromagnetic and antiferromagnetic modes in c-cut and b-cut YFeO_3_ crystals using two THz pulses [183]. By adjusting the delay time of the THz pulse pair, it is possible to selectively enhance or diminish a certain spin mode. Then, these authors demonstrated the energy transfer between spin waves and photon spin systems in dual-pulse coherent control [184]. A single pulse to achieve coherent control is based on the birefringence effect of the material [185].

**Figure 16:**
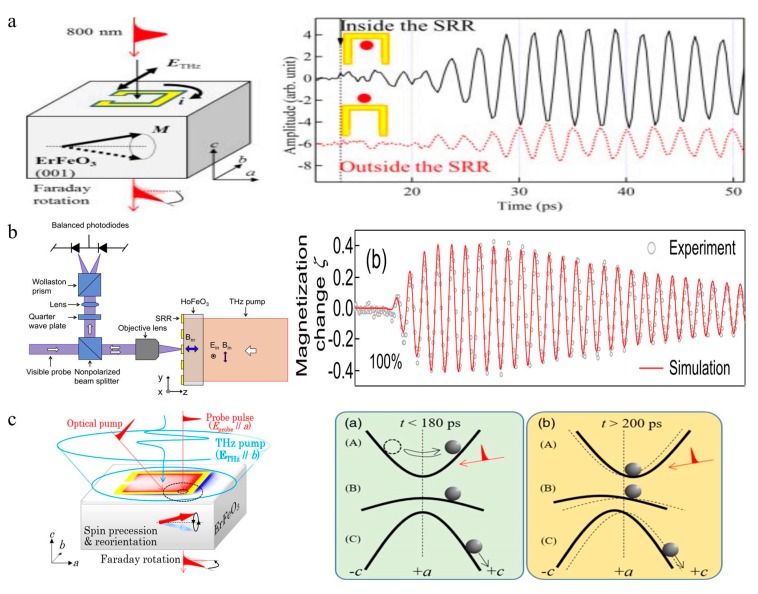
(**a**) Exciting the antiferromagnetic mode using the terahertz magnetic field of the SRR. The right figure shows that the spin precession measures the internal (black solid curve) and the outer (red dotted curve) SRR. Obvious 180 phase differences and amplitude changes were observed [186], copyright IEEE, 2012. (**b**) Schematic setup of THz pump-visible MOKE measurements. The relationship between the magnetization change and time is obtained by experiment and simulation [187]. (**c**) Experimental configuration using the SRR structure. The right side shows the dominant process of symmetry destruction in the terahertz magnetic field. The magnetization tilt is caused by the free spin precession and non-resonant field excitation forced oscillation [188], copyright American Physical Society, 2018.

Based on the above mentioned studies, considering that an SRR can generate a circulating current by an LC circuit that is excited by the electric field of a THz wave to generate a magnetic field, the combination of metamaterials and an antiferromagnet will increase the controllability of the spin wave. In 2014, Kurihara et al. demonstrated the resonant excitation of the spin precession of ErFeO_3_ with a magnetic field produced by the SRR [186], as shown in Figure 16a. When the resonance frequency of SRR was similar to the resonance frequency of the spin precession, the amplitude of the spin wave was greatly increased. The SRR resonant magnetic field was 20 times stronger than the incident THz-pulsed magnetic field [173]. Mukai et al. reported on the nonlinear magnetization dynamics of HoFeO_3_ crystals based on the strong terahertz magnetic field of a split-ring resonator (Figure 16b) [187]. A strong THz magnetic field can cause a large magnetization change of 40%, and the change in magnetization can remain sufficiently large to cause a redshift even after the magnetic field disappeared. Kurihara et al. demonstrated a combination of the terahertz magnetic field of the SRR and femtosecond laser excitation to break the symmetry of the light-induced spin reorientation path in ErFeO_3_ [188]. By controlling the arrival time of the optical and terahertz pump pulses, the final state reaches more than 80% of the total magnetization in the optional direction. The strong magnetic field provided by metamaterials can bring a new approach to the study of antiferromagnets (Figure 16c).

In addition to the magnetic field, ferrous materials also play an important role in modulating electromagnetic waves through an electric field. The permittivity of the ferroelectric material can be adjusted by DC electric field, thereby modulating the phase of the electromagnetic wave. Yin et al. designed a metasurface based on a resonator of ferroelectric material that enables adjustment of the resonant phase of the resonator over a dynamic range [189]. The permittivity of the ferroelectric material is varied by applied electric field, causing a phase shift of the reflected wave, up to 2π. This resonator can be used as a planar lens to focus the reflected wave. Yu et al. studied the dielectric properties of barium titanate ferroelectric thin films by illumination and found that the permittivity increases with the increase of optical power [21]. The device can achieve a modulation depth of 40% at 0.2 THz by varying the illumination power.

## 9. Conclusions

Dynamically tunable metasurfaces based on different functional materials can offer various functionalities in manipulating the spectral and spatial characteristics of terahertz waves to form different types of modulators, such as amplitude modulators, phase modulators, polarization modulators, and programmable modulators. In this article, we have briefly reviewed the latest developments of THz modulators with dynamic tunable metasurfaces based on representative materials. As the active elements and the performance of the THz modulator greatly depend on the material, each category of dynamic tunable metasurfaces was organized and discussed according to the material characteristics. For the THz modulators, high modulation speed and high efficiency are the eternal goals. Thus, we found that many outstanding studies have focused on improving the modulation speed, modulation depth and phase. During the past 10 years, the development of active metasurfaces has promoted the performance of THz modulators to a high level. The modulation speed has been improved from kHz to GHz, the modulation depth has reached nearly 100%, the phase modulation has broken through 130° in the transmission mode, and new kinds of modulators, such as on-chip modulators and compressing sensor modulators, have been proposed. Such achievements of THz modulators with dynamic tunable metasurfaces have already offered unprecedented functionality for manipulating THz waves.

However, until now, the existing performance of the THz modulator cannot satisfy the need for the practical application systems. Most of the THz modulators remain in the laboratory or research settings. It is difficult to achieve a balance of high resolution and real-time imaging for modulators, especially in complex imaging scenes. Additionally, modulators with modulation speed higher than 10 Gbps are necessary for practical high-speed communication systems, and transmissive phase modulators currently cannot achieve a phase modulation of 2π, which cannot satisfy the need of practical applications. Large arrays of terahertz spatial modulators will inevitably face the problem of monolithic integrated matching circuits and the like. In the future, new resonant mechanisms, diverse modulation methods and the design of the new resonant structures are expected to further improve the performance of the terahertz modulators. Due to the maturity of semiconductor materials in the fabrication process, we believe materials, such as graphene, 2DEG materials, superconducting materials, vanadium, etc., with excellent characteristics will facilitate a bright future for the THz modulators. With rapid progress in the terahertz field and the development of high-performance THz applications, we envision that coding metasurfaces, miniaturized or on-chip active metasurfaces, and hook face active metasurfaces will be developed and THz modulators will play a key role in THz systems.

## Figures and Tables

**Table 1:** THz amplitude modulator type, materials, and basic performance.

Type	Material	Mod Speed or Response Time	Mod Depth	Ref.
EC MS	GaAs	2 MHz	55%	2009 [9]
EC MS	GaAs	ps range	100%	2019 [10]
PI MS	Silicon	20/300 ps	50%	2018 [11]
2D MS	Graphene	100 MHz	25%	2016 [12]
2D MS	MoS2	100 ps	100%	2017 [13]
2DEGs MS	GaN HEMT	1 GHz	85%	2015 [14]
2DEGs MS	GaAs HEMT	2.7 MHz	80%	2017 [15]
VO_2_ MS	VO_2_	1 MHz	88%	2014 [16]
Liquid Crystal MS	Liquid Crystal	50 ms	100%	2015 [17]
Liquid Crystal MS	Liquid Crystal	\	80%	2017 [18]
Superconducting MS	YBCO	80 ps	86%/60%	2018 [19]
Superconducting MS	NbN	1 MHz	79.8%	2017 [20]
Spintronics MS	BaTiO3	\	40%	2017 [21]

MS, Metasurface; EC, Electrical controlled MS; PI, Photo-induced MS; 2D, Two-dimensional material; 2DEGs, Two-dimensional electron gas.

**Table 2:** THz phase modulator type, materials, and basic performances.

Type	Material	Mod Speed	Phase Mod	Ref
EC MS	GaAs	>2 MHz	0.56 rad	2009 [9]
PI MS	GaAs	—	0.78 rad	2010 [22]
2D MS	Graphene	100 kHz	0.56 rad	2012 [23]
2D MS	Graphene	20 kHz	0.73 rad	2014 [24]
2DEGs MS	GaAs HEMT	2.7 MHz	0.67 rad	2017 [15]
2DEGs MS	GaN HEMT	__	2.39 rad	2018 [25]
VO2 MS	VO2	—	1.02 rad	2016 [26]
VO2 MS	VO2	__	2.41 rad	2018 [27]
Liquid Crystal MS	Liquid crystal	__	1.03 rad	2017 [28]

MS, Metasurface; EC, Electrical controlled MS; PI, Photo-induced MS; 2D, Two-dimensional material; 2DEGs, Two-dimensional electron gas.

## References

[1] Rahm M., Li J.S., Padilla W.J. (2013). THz wave modulators: A brief review on different modulation techniques. J. Infrared Millim. Terahertz Waves.

[2] Li A.B., Singh S., Sievenpiper D. (2018). Metasurfaces and their applications. Nanophotonics.

[3] Yu N.F., Genevet P., Kats M.A., Aieta F., Tetienne J.P., Capasso F., Gaburro Z. (2011). Light propagation with phase discontinuities: Generalized laws of reflection and refraction. Science.

[4] Genevet P., Yu N.F., Aieta F., Lin J., Kats M.A., Blanchard R., Scully M.O., Gaburro Z., Capasso F. (2012). Ultra-thin plasmonic optical vortex plate based on phase discontinuities. Appl. Phys. Lett..

[5] Yu N.F., Aieta F., Genevet P., Kats M.A., Gaburro Z., Capasso F. (2012). A broadband, background-free quarter-wave plate based on plasmonic metasurfaces. Nano Lett..

[6] Huang W.X., Zhang Y., Tang X.M., Cai L.S., Zhao J.W., Zhou L., Wang Q.J., Huang C.P., Zhu Y.Y. (2011). Optical properties of a planar metamaterial with chiral symmetry breaking. Opt. Lett..

[7] Shirmanesh G.K., Sokhoyan R., Pala R.A., Atwater H.A. (2018). Dual-gated active metasurface at 1550 nm with wide (> 300°) phase tunability. Nano Lett..

[8] Hum S.V., Okoniewski M., Davies R.J. (2007). Modeling and design of electronically tunable reflectarrays. IEEE Trans. Antennas Propag..

[9] Chen H.-T., Padilla W.J., Cich M.J., Azad A.K., Averitt R.D., Taylor A.J. (2009). A metamaterial solid-state terahertz phase modulator. Nat. Photonics.

[10] Isic G., Sinatkas G., Zografopoulos D.C., Vasic B., Ferraro A., Beccherelli R., Kriezis E.E., Belic M. (2019). Electrically tunable metal-semiconductor-metal terahertz metasurface modulators. IEEE J. Sel. Top. Quant..

[11] Shen X.P., Cui T.J. (2012). Photoexcited broadband redshift switch and strength modulation of terahertz metamaterial absorber. J. Opt..

[12] Jessop D.S., Kindness S.J., Xiao L., Braeuninger-Weimer P., Lin H., Ren Y., Ren C.X., Hofmann S., Zeitler J.A., Beere H.E. (2016). Graphene based plasmonic terahertz amplitude modulator operating above 100 MHz. Appl. Phys. Lett..

[13] Srivastava Y.K., Chaturvedi A., Manjappa M., Kumar A., Dayal G., Kloc C., Singh R. (2017). MoS2 for ultrafast all-optical switching and modulation of THz fano metaphotonic devices. Adv. Opt. Mater..

[14] Zhang Y., Qiao S., Liang S., Wu Z., Yang Z., Feng Z., Sun H., Zhou Y., Sun L., Chen Z. (2015). Gbps terahertz external modulator based on a composite metamaterial with a double-channel heterostructure. Nano Lett..

[15] Zhou Z., Wang S., Yu Y., Chen Y., Feng L. (2017). High performance metamaterials-high electron mobility transistors integrated terahertz modulator. Opt. Express.

[16] Zhang Y.X., Qiao S., Sun L.L., Shi Q.W., Huang W.X., Li L., Yang Z.Q. (2014). Photoinduced active terahertz metamaterials with nanostructured vanadium dioxide film deposited by sol-gel method. Opt. Express.

[17] Isic G., Vasic B., Zografopoulos D.C., Beccherelli R., Gajic R. (2015). Electrically tunable critically coupled terahertz metamaterial absorber based on nematic liquid crystals. Phys. Rev. Appl..

[18] Wang L., Ge S., Hu W., Nakajima M., Lu Y. (2017). Graphene-assisted high-efficiency liquid crystal tunable terahertz metamaterial absorber. Opt. Express.

[19] Srivastava Y.K., Manjappa M., Cong L.Q., Krishnamoorthy H.N.S., Savinov V., Pitchappa P., Singh R. (2018). A superconducting dual-channel photonic switch. Adv. Mater..

[20] Li C., Wu J.B., Jiang S.L., Su R.F., Zhang C.H., Jiang C.T., Zhou G.C., Jin B.B., Kang L., Xu W.W. (2017). Electrical dynamic modulation of THz radiation based on superconducting metamaterials. Appl. Phys. Lett..

[21] Zhou S.Y., Ji J., Tian Y., Ling F., Yu W.F. (2017). Optically tuned dielectric property of barium titanate thin film by THz spectroscopy. Opt. Mater..

[22] Manceau J.M., Shen N.H., Kafesaki M., Soukoulis C.M., Tzortzakis S. (2010). Dynamic response of metamaterials in the terahertz regime: Blueshift tunability and broadband phase modulation. Appl. Phys. Lett..

[23] Lee S.H., Choi M., Kim T.T., Lee S., Liu M., Yin X., Choi H.K., Lee S.S., Choi C.G., Choi S.Y. (2012). Switching terahertz waves with gate-controlled active graphene metamaterials. Nat. Mater..

[24] Arezoomandan S., Yang K., Sensale-Rodriguez B. (2014). Graphene-based electrically reconfigurable deep-subwavelength metamaterials for active control of THz light propagation. Appl. Phys. A-Mater..

[25] Zhang Y., Zhao Y., Liang S., Zhang B., Wang L., Zhou T., Kou W., Lan F., Zeng H., Han J. (2018). Large phase modulation of THz wave via an enhanced resonant active HEMT metasurface. Nanophotonics.

[26] Hashemi M.R.M., Yang S.H., Wang T.Y., Sepulveda N., Jarrahi M. (2016). Electronically-controlled beam-steering through vanadium dioxide metasurfaces. Sci. Rep..

[27] Zhao Y.C., Zhang Y.X., Shi Q.W., Liang S.X., Huang W.X., Kou W., Yang Z.Q. (2018). Dynamic photoinduced controlling of the large phase shift of terahertz waves via vanadium dioxide coupling nanostructures. ACS Photonics.

[28] Ji Y.Y., Fan F., Chen M., Yang L., Chang S.J. (2017). Terahertz artificial birefringence and tunable phase shifter based on dielectric metasurface with compound lattice. Opt. Express.

[29] Liu F., Pitilakis A., Mirmoosa M.S., Tsilipakos O., Wang X.C., Tasolamprou A.C., Abadal S., Cabellos-Aparicio A., Alarcon E., Liaskos C. Programmable metasurfaces: State of the art and prospects. Proceedings of the 2018 IEEE International Symposium on Circuits and Systems.

[30] Cui T., Bai B.F., Sun H.B. (2019). Tunable metasurfaces based on active materials. Adv. Funct. Mater..

[31] Degl’Innocenti R., Kindness S.J., Beere H.E., Ritchie D.A. (2018). All-integrated terahertz modulators. Nanophotonics.

[32] Ding F., Yang Y.Q., Deshpande R.A., Bozhevolnyi S.I. (2018). A review of gap-surface plasmon metasurfaces: Fundamentals and applications. Nanophotonics.

[33] Cheng J.R., Fan F., Chang S.J. (2019). Recent progress on graphene-functionalized metasurfaces for tunable Phase and polarization control. Nanomaterials.

[34] Chen H.T., Padilla W.J., Zide J.M., Gossard A.C., Taylor A.J., Averitt R.D. (2006). Active terahertz metamaterial devices. Nature.

[35] Paul O., Imhof C., Lagel B., Wolff S., Heinrich J., Hofling S., Forchel A., Zengerle R., Beigang R., Rahm M. (2009). Polarization-independent active metamaterial for high-frequency terahertz modulation. Opt. Express.

[36] Jun Y.C., Gonzales E., Reno J.L., Shaner E.A., Gabbay A., Brener I. (2012). Active tuning of mid-infrared metamaterials by electrical control of carrier densities. Opt. Express.

[37] Liao M.L., Cong J.W., Zhang X., Cui Y.P. (2013). Development of an electrically controlled terahertz-wave modulator. J. Mod. Opt..

[38] Karl N., Reichel K., Chen H.T., Taylor A.J., Brener I., Benz A., Reno J.L., Mendis R., Mittleman D.M. (2014). An electrically driven terahertz metamaterial diffractive modulator with more than 20 dB of dynamic range. Appl. Phys. Lett..

[39] Keiser G.R., Karl N., Liu P.Q., Tulloss C., Chen H.T., Taylor A.J., Brener I., Reno J.L., Mittleman D.M. (2017). Nonlinear terahertz metamaterials with active electrical control. Appl. Phys. Lett..

[40] Bai Y., Chen K.J., Bu T., Zhuang S.L. (2016). An electrically tunable terahertz metamaterial modulator with two independent channels. J. Appl. Phys..

[41] Li J.S. (2011). Terahertz wave modulator based on optically controllable metamaterial. Opt. Laser Technol..

[42] Shen N.H., Massaouti M., Gokkavas M., Manceau J.M., Ozbay E., Kafesaki M., Koschny T., Tzortzakis S., Soukoulis C.M. (2011). Optically implemented broadband blueshift switch in the terahertz regime. Phys. Rev. Lett..

[43] Seren H.R., Keiser G.R., Cao L.Y., Zhang J.D., Strikwerda A.C., Fan K.B., Metcalfe G.D., Wraback M., Zhang X., Averitt R.D. (2014). Optically modulated multiband terahertz perfect absorber. Adv. Opt. Mater..

[44] Deng L.Y., Teng J.H., Liu H.W., Wu Q.Y., Tang J., Zhang X.H., Maier S.A., Lim K.P., Ngo C.Y., Yoon S.F. (2013). Direct optical tuning of the terahertz plasmonic response of InSb subwavelength gratings. Adv. Opt. Mater..

[45] Yang Y.M., Kamaraju N., Campione S., Liu S., Reno J.L., Sinclair M.B., Prasankumar R.P., Brener I. (2017). Transient GaAs plasmonic metasurfaces at terahertz frequencies. ACS Photonics.

[46] Shcherbakov M.R., Liu S., Zubyuk V.V., Vaskin A., Vabishchevich P.P., Keeler G., Pertsch T., Dolgova T.V., Staude I., Brener I. (2017). Ultrafast all-optical tuning of direct-gap semiconductor metasurfaces. Nat. Commun..

[47] Cai H.L., Huang Q.P., Hu X., Liu Y., Fu Z.P., Zhao Y., He H.C., Lu Y.L. (2018). All-optical and ultrafast tuning of terahertz plasmonic metasurfaces. Adv. Opt. Mater..

[48] Wang B., Dong F.L., Li Q.T., Yang D., Sun C.W., Chen J.J., Song Z.W., Xu L.H., Chu W.G., Xiao Y.F. (2016). Visible-frequency dielectric metasurfaces for multiwavelength achromatic and highly dispersive holograms. Nano Lett..

[49] Kamaraju N., Rubano A., Jian L.K., Saha S., Venkatesan T., Notzold J., Campen R.K., Wolf M., Kampfrath T. (2014). Subcycle control of terahertz waveform polarization using all-optically induced transient metamaterials. Light-Sci. Appl..

[50] Seren H.R., Zhang J.D., Keiser G.R., Maddox S.J., Zhao X.G., Fan K.B., Bank S.R., Zhang X., Averitt R.D. (2016). Nonlinear terahertz devices utilizing semiconducting plasmonic metamaterials. Light-Sci. Appl..

[51] Cui T.J., Qi M.Q., Wan X., Zhao J., Cheng Q. (2014). Coding metamaterials, digital metamaterials and programmable metamaterials. Light-Sci. Appl..

[52] Gao L.H., Cheng Q., Yang J., Ma S.J., Zhao J., Liu S., Chen H.B., He Q., Jiang W.X., Ma H.F. (2015). Broadband diffusion of terahertz waves by multi-bit coding metasurfaces. Light-Sci. Appl..

[53] Li L.L., Cui T.J., Ji W., Liu S., Ding J., Wan X., Li Y.B., Jiang M.H., Qiu C.W., Zhang S. (2017). Electromagnetic reprogrammable coding-metasurface holograms. Nat. Commun..

[54] Zhang L., Liu S., Li L.L., Cui T.J. (2017). Spin-controlled multiple pencil beams and vortex beams with different polarizations generated by pancharatnam-berry coding metasurfaces. ACS Appl. Mater. Interfaces.

[55] Ma Q., Shi C.B., Bai G.D., Chen T.Y., Noor A., Cui T.J. (2017). Beam-editing coding metasurfaces based on polarization bit and orbital-angular-momentum-mode bit. Adv. Opt. Mater..

[56] Liu S., Zhang L., Yang Q.L., Xu Q., Yang Y., Noor A., Zhang Q., Iqbal S., Wan X., Tian Z. (2016). Frequency-dependent dual-functional coding metasurfaces at terahertz frequencies. Adv. Opt. Mater..

[57] Liu S., Zhang H.C., Zhang L., Yang Q.L., Xu Q., Gu J.Q., Yang Y., Zhou X.Y., Han J.G., Cheng Q. (2017). Full-state controls of terahertz waves using tensor coding metasurfaces. ACS Appl. Mater. Interfaces.

[58] Liu S., Noor A., Du L.L., Zhang L., Xu Q., Luan K., Wang T.Q., Tian Z., Tang W.X., Han J.G. (2016). Anomalous refraction and nondiffractive bessel-beam generation of terahertz waves through transmission-type coding metasurfaces. ACS Photonics.

[59] Huang C., Sun B., Pan W.B., Cui J.H., Wu X.Y., Luo X.G. (2017). Dynamical beam manipulation based on 2-bit digitally-controlled coding metasurface. Sci. Rep..

[60] Yang H.H., Cao X.Y., Yang F., Gao J., Xu S.H., Li M.K., Chen X.B., Zhao Y., Zheng Y.J., Li S.J. (2016). A programmable metasurface with dynamic polarization, scattering and focusing control. Sci. Rep..

[61] Zhang L., Wan X., Liu S., Yin J.Y., Zhang Q., Wu H.T., Cui T.J. (2017). Realization of low scattering for a high-gain Fabry-Perot antenna using coding metasurface. IEEE Trans. Antennas Propag..

[62] Moccia M., Liu S., Wu R.Y., Castaldi G., Andreone A., Cui T.J., Galdi V. (2017). Coding Metasurfaces for diffuse scattering: Scaling laws, bounds, and suboptimal design. Adv. Opt. Mater..

[63] Zhang L., Chen X.Q., Liu S., Zhang Q., Zhao J., Dai J.Y., Bai G.D., Wan X., Cheng Q., Castaldi G. (2018). Space-time-coding digital metasurfaces. Nat. Commun..

[64] Candes E.J., Wakin M.B. (2008). An introduction to compressive sampling. IEEE Signal Proc. Mag..

[65] Takhar D., Laska J.N., Wakin M.B., Duarte M.E., Baron D., Sarvotham S., Kelly K.E., Baraniuk R.G., Bouman C.A., Miller E.L., Pollak I. (2006). A new Compressive Imaging camera architecture using optical-domain compression. Computational Imaging IV.

[66] Watts C.M., Shrekenhamer D., Montoya J., Lipworth G., Hunt J., Sleasman T., Krishna S., Smith D.R., Padilla W.J. (2014). Terahertz compressive imaging with metamaterial spatial light modulators. Nat. Photonics.

[67] Stantchev R.I., Phillips D.B., Hobson P., Hornett S.M., Padgett M.J., Hendry E. (2017). Compressed sensing with near-field THz radiation. Optica.

[68] Yamagiwa M., Ogawa T., Minamikawa T., Abdelsalam D.G., Okabe K., Tsurumachi N., Mizutani Y., Iwata T., Yamamoto H., Yasui T. (2018). Real-time amplitude and phase imaging of optically opaque objects by combining full-field off-axis terahertz digital holography with angular spectrum reconstruction. J. Infrared Millim. Terahertz Waves.

[69] Zheng G.X., Muhlenbernd H., Kenney M., Li G.X., Zentgraf T., Zhang S. (2015). Metasurface holograms reaching 80% efficiency. Nat. Nanotechnol..

[70] Wen D.D., Yue F.Y., Li G.X., Zheng G.X., Chan K.L., Chen S.M., Chen M., Li K.F., Wong P.W.H., Cheah K.W. (2015). Helicity multiplexed broadband metasurface holograms. Nat. Commun..

[71] Wang Q., Xu Q., Zhang X.Q., Tian C.X., Xu Y.H., Gu J.Q., Tian Z., Ouyang C.M., Zhang X.X., Han J.G. (2018). All-dielectric meta-holograms with holographic images transforming longitudinally. ACS Photonics.

[72] Guo J., Wang T., Zhao H., Wang X., Feng S., Han P., Sun W., Ye J., Situ G., Chen H.T. (2019). Reconfigurable terahertz metasurface pure phase holograms. Adv. Opt. Mater..

[73] Zhao H., Wang X., He J., Guo J., Ye J., Kan Q., Zhang Y. (2017). High-efficiency terahertz devices based on cross-polarization converter. Sci Rep..

[74] Wang Q., Plum E., Yang Q.L., Zhang X.Q., Xu Q., Xu Y.H., Han J.G., Zhang W.L. (2018). Reflective chiral meta-holography: Multiplexing holograms for circularly polarized waves. Light-Sci. Appl..

[75] Sinyukov A.M., Liu Z.W., Hor Y.L., Su K., Barat R.B., Gary D.E., Michalopoulou Z.H., Zorych I., Federici J.F., Zimdars D. (2008). Rapid-phase modulation of terahertz radiation for high-speed terahertz imaging and spectroscopy. Opt. Lett..

[76] Chan W.L., Chen H.T., Taylor A.J., Brener I., Cich M.J., Mittleman D.M. (2009). A spatial light modulator for terahertz beams. Appl. Phys. Lett..

[77] Eastman L.F., Tilak V., Smart J., Green B.M., Chumbes E.M., Dimitrov R., Kim H., Ambacher O.S., Weimann N., Prunty T. (2001). Undoped AlGaN/GaN HEMTs for microwave power amplification. IEEE T. Electron. Dev..

[78] Shrekenhamer D., Rout S., Strikwerda A.C., Bingham C., Averitt R.D., Sonkusale S., Padilla W.J. (2011). High speed terahertz modulation from metamaterials with embedded high electron mobility transistors. Opt. Express.

[79] Huang Y.D., Yu Y., Qin H., Sun J.D., Zhang Z.P., Li X.X., Huang J.J., Cai Y. (2016). Plasmonic terahertz modulator based on a grating-coupled two-dimensional electron system. Appl. Phys. Lett..

[80] Lee G., Nouman M.T., Hwang J.H., Kim H.W., Jang J.H. (2018). Enhancing the modulation depth of a dynamic terahertz metasurface by integrating into an asymmetric Fabry-Pérot cavity. AIP Adv..

[81] Zhang X., Xing Y., Zhang Q., Gu Y., Su Y., Ma C. (2018). High speed terahertz modulator based on the single channel AlGaN/GaN high electron mobility transistor. Solid State Electron..

[82] Nouman M.T., Kim H.W., Woo J.M., Hwang J.H., Kim D., Jang J.H. (2016). Terahertz modulator based on metamaterials integrated with metal-semiconductor-metal varactors. Sci Rep..

[83] Rout S., Sonkusale S.R. (2016). A low-voltage high-speed terahertz spatial light modulator using active metamaterial. APL Photonics.

[84] Novoselov K.S., Geim A.K., Morozov S.V., Jiang D., Zhang Y., Dubonos S.V., Grigorieva I.V., Firsov A.A. (2004). Electric field effect in atomically thin carbon films. Science.

[85] Ferrari A.C., Bonaccorso F., Fal’ko V., Novoselov K.S., Roche S., Boggild P., Borini S., Koppens F.H.L., Palermo V., Pugno N. (2015). Science and technology roadmap for graphene, related two-dimensional crystals, and hybrid systems. Nanoscale.

[86] Sun Z.P., Martinez A., Wang F. (2016). Optical modulators with 2D layered materials. Nat. Photonics.

[87] Xia F.N., Wang H., Xiao D., Dubey M., Ramasubramaniam A. (2014). Two-dimensional material nanophotonics. Nat. Photonics.

[88] Boyd D.A., Lin W.H., Hsu C.C., Teague M.L., Chen C.C., Lo Y.Y., Chan W.Y., Su W.B., Cheng T.C., Chang C.S. (2015). Single-step deposition of high-mobility graphene at reduced temperatures. Nat. Commun..

[89] Efetov D.K., Kim P. (2010). Controlling electron-phonon interactions in graphene at ultrahigh carrier densities. Phys. Rev. Lett..

[90] Sensale-Rodriguez B., Yan R.S., Kelly M.M., Fang T., Tahy K., Hwang W.S., Jena D., Liu L., Xing H.G. (2012). Broadband graphene terahertz modulators enabled by intraband transitions. Nat. Commun..

[91] Sensale-Rodriguez B., Yan R.S., Rafique S., Zhu M.D., Li W., Liang X.L., Gundlach D., Protasenko V., Kelly M.M., Jena D. (2012). Extraordinary control of terahertz beam reflectance in graphene electro-absorption modulators. Nano Lett..

[92] Valmorra F., Scalari G., Maissen C., Fu W.Y., Schonenberger C., Choi J.W., Park H.G., Beck M., Faist J. (2013). Low-bias active control of terahertz waves by coupling large-area CVD graphene to a terahertz metamaterial. Nano Lett..

[93] Degl’Innocenti R., Jessop D.S., Shah Y.D., Sibik J., Zeitler J.A., Kidambi P.R., Hofmann S., Beere H.E., Ritchie D.A. (2014). Low-Bias Terahertz Amplitude Modulator Based on Split-Ring Resonators and Graphene. ACS Nano.

[94] Chen Z.F., Chen X.Q., Tao L., Chen K., Long M.Z., Liu X.D., Yan K.Y., Stantchev R.I., Pickwell-MacPherson E., Xu J.B. (2018). Graphene controlled Brewster angle device for ultra broadband terahertz modulation. Nat. Commun..

[95] Li Q., Tian Z., Zhang X.Q., Singh R., Du L.L., Gu J.Q., Han J.G., Zhang W.L. (2015). Active graphene-silicon hybrid diode for terahertz waves. Nat. Commun..

[96] Liang G.Z., Hu X.N., Yu X.C., Shen Y.D., Li L.H.H., Davies A.G., Linfield E.H., Liang H.K., Zhang Y., Yu S.F. (2015). Integrated terahertz graphene modulator with 100% modulation depth. ACS Photonics.

[97] Sensale-Rodriguez B., Rafique S., Yan R.S., Zhu M.D., Protasenko V., Jena D., Liu L., Xing H.G. (2013). Terahertz imaging employing graphene modulator arrays. Opt. Express.

[98] Sensale-Rodriguez B., Yan R.S., Zhu M.D., Jena D., Liu L., Xing H.G. (2012). Efficient terahertz electro-absorption modulation employing graphene plasmonic structures. Appl. Phys. Lett..

[99] Liu P.Q., Luxmoore I.J., Mikhailov S.A., Savostianova N.A., Valmorra F., Faist J., Nash G.R. (2015). Highly tunable hybrid metamaterials employing split-ring resonators strongly coupled to graphene surface plasmons. Nat. Commun..

[100] Ju L., Geng B.S., Horng J., Girit C., Martin M., Hao Z., Bechtel H.A., Liang X.G., Zettl A., Shen Y.R. (2011). Graphene plasmonics for tunable terahertz metamaterials. Nat. Nanotechnol..

[101] Gao W.L., Shu J., Reichel K., Nickel D.V., He X.W., Shi G., Vajtai R., Ajayan P.M., Kono J., Mittleman D.M. (2014). High-contrast terahertz wave modulation by gated graphene enhanced by extraordinary transmission through ring apertures. Nano Lett..

[102] Papasimakis N., Luo Z.Q., Shen Z.X., De Angelis F., Di Fabrizio E., Nikolaenko A.E., Zheludev N.I. (2010). Graphene in a photonic metamaterial. Opt. Express.

[103] Yao Y., Shankar R., Kats M.A., Song Y., Kong J., Loncar M., Capasso F. (2014). Electrically tunable metasurface perfect absorbers for ultrathin mid-infrared optical modulators. Nano Lett..

[104] Yan H.G., Li X.S., Chandra B., Tulevski G., Wu Y.Q., Freitag M., Zhu W.J., Avouris P., Xia F.N. (2012). Tunable infrared plasmonic devices using graphene/insulator stacks. Nat. Nanotechnol..

[105] Liu M., Yin X., Ulin-Avila E., Geng B., Zentgraf T., Ju L., Wang F., Zhang X. (2011). A graphene-based broadband optical modulator. Nature.

[106] Dalir H., Xia Y., Wang Y., Zhang X. (2016). Athermal broadband graphene optical modulator with 35 GHz speed. ACS Photonics.

[107] Kim J., Son H., Cho D.J., Geng B.S., Regan W., Shi S.F., Kim K., Zettl A., Shen Y.R., Wang F. (2012). Electrical control of optical plasmon resonance with graphene. Nano Lett..

[108] Emani N.K., Chung T.F., Ni X.J., Kildishev A.V., Chen Y.P., Boltasseva A. (2012). Electrically tunable damping of plasmonic resonances with graphene. Nano Lett..

[109] Searles T.A., Rezaee M., Strickland E., Brower-Thomas T.L., Harris G.L., Yahiaoui R., Lue W. Graphene-based metasurfaces for multimode tunable terahertz modulators. Proceedings of the Conference on Lasers and Electro-Optics.

[110] Li W., Chen B.G., Meng C., Fang W., Xiao Y., Li X.Y., Hu Z.F., Xu Y.X., Tong L.M., Wang H.Q. (2014). Ultrafast all-optical graphene modulator. Nano Lett..

[111] Yu S.L., Wu X.Q., Chen K.R., Chen B.G., Guo X., Dai D.X., Tong L.M., Liu W.T., Shen Y.R. (2016). All-optical graphene modulator based on optical Kerr phase shift. Optica.

[112] Weis P., Garcia-Pomar J.L., Hoh M., Reinhard B., Brodyanski A., Rahm M. (2012). Spectrally wide-band terahertz wave modulator based on optically tuned graphene. ACS Nano.

[113] Novoselov K.S., Jiang D., Schedin F., Booth T.J., Khotkevich V.V., Morozov S.V., Geim A.K. (2005). Two-dimensional atomic crystals. Proc. Natl. Acad. Sci. USA.

[114] Mak K.F., Lee C., Hone J., Shan J., Heinz T.F. (2010). Atomically thin MoS2: A new direct-gap semiconductor. Phys. Rev. Lett..

[115] Splendiani A., Sun L., Zhang Y.B., Li T.S., Kim J., Chim C.Y., Galli G., Wang F. (2010). Emerging photoluminescence in monolayer MoS2. Nano Lett..

[116] Li L.K., Yu Y.J., Ye G.J., Ge Q.Q., Ou X.D., Wu H., Feng D.L., Chen X.H., Zhang Y.B. (2014). Black phosphorus field-effect transistors. Nat. Nanotechnol..

[117] Manzeli S., Ovchinnikov D., Pasquier D., Yazyev O.V., Kis A. (2017). 2D transition metal dichalcogenides. Nat. Rev. Mater..

[118] Chen S., Fan F., Miao Y.P., He X.T., Zhang K.L., Chang S.J. (2016). Ultrasensitive terahertz modulation by silicon-grown MoS2 nanosheets. Nanoscale.

[119] Cao Y.P., Gan S., Geng Z.X., Liu J., Yang Y.P., Bao Q.L., Chen H.D. (2016). Optically tuned terahertz modulator based on annealed multilayer MoS2. Sci. Rep..

[120] Morin F.J. (1959). Oxides which show a metal-to-insulator transition at the neel temperature. Phys. Rev. Lett..

[121] Cavalleri A., Toth C., Siders C.W., Squier J.A., Raksi F., Forget P., Kieffer J.C. (2001). Femtosecond structural dynamics in VO_2_ during an ultrafast solid-solid phase transition. Phys. Rev. Lett..

[122] Kubler C., Ehrke H., Huber R., Lopez R., Halabica A., Haglund R.F., Leitenstorfer A. (2007). Coherent structural dynamics and electronic correlations during an ultrafast insulator-to-metal phase transition in VO_2_. Phys. Rev. Lett..

[123] Jeong J., Aetukuri N., Graf T., Schladt T.D., Samant M.G., Parkin S.S.P. (2013). Suppression of metal-insulator transition in VO_2_ by electric field-induced oxygen vacancy formation. Science.

[124] Driscoll T., Kim H.T., Chae B.G., Kim B.J., Lee Y.W., Jokerst N.M., Palit S., Smith D.R., Di Ventra M., Basov D.N. (2009). Memory metamaterials. Science.

[125] Liu M.K., Hwang H.Y., Tao H., Strikwerda A.C., Fan K.B., Keiser G.R., Sternbach A.J., West K.G., Kittiwatanakul S., Lu J.W. (2012). Terahertz-field-induced insulator-to-metal transition in vanadium dioxide metamaterial. Nature.

[126] Wen Q.Y., Zhang H.W., Yang Q.H., Xie Y.S., Chen K., Liu Y.L. (2010). Terahertz metamaterials with VO_2_ cut-wires for thermal tunability. Appl. Phys. Lett..

[127] Seo M., Kyoung J., Park H., Koo S., Kim H.S., Bernien H., Kim B.J., Choe J.H., Ahn Y.H., Kim H.T. (2010). Active terahertz nanoantennas based on VO_2_ phase transition. Nano Lett..

[128] Jeong Y.G., Bernien H., Kyoung J.S., Park H.R., Kim H.S., Choi J.W., Kim B.J., Kim H.T., Ahn K.J., Kim D.S. (2011). Electrical control of terahertz nano antennas on VO_2_ thin film. Opt. Express.

[129] Zhu Y.H., Vegesna S., Zhao Y., Kuryatkov V., Holtz M., Fan Z.Y., Saed M., Bernussi A.A. (2013). Tunable dual-band terahertz metamaterial bandpass filters. Opt. Lett..

[130] Zhou G.C., Dai P.H., Wu J.B., Jin B.B., Wen Q.Y., Zhu G.H., Shen Z., Zhang C.H., Kang L., Xu W.W. (2017). Broadband and high modulation-depth THz modulator using low bias controlled VO_2_-integrated metasurface. Opt. Express.

[131] Liu L., Kang L., Mayer T.S., Werner D.H. (2016). Hybrid metamaterials for electrically triggered multifunctional control. Nat. Commun..

[132] Cai H.L., Chen S., Zou C.W., Huang Q.P., Liu Y., Hu X., Fu Z.P., Zhao Y., He H.C., Lu Y.L. (2018). Multifunctional hybrid metasurfaces for dynamic tuning of terahertz waves. Adv. Opt. Mater..

[133] Shin J.H., Han S.P., Song M., Ryu H.C. (2019). Gradual tuning of the terahertz passband using a square-loop metamaterial based on a W-doped VO_2_ thin film. Appl. Phys. Express.

[134] Fan F., Hou Y., Jiang Z.W., Wang X.H., Chang S.J. (2012). Terahertz modulator based on insulator-metal transition in photonic crystal waveguide. Appl. Opt..

[135] Zhang K., Zhang L., Duan D., Fan Y.X., Tao Z.Y. (2018). Wide band terahertz switch of undulated waveguide with VO_2_ film coated inner wall. J. Lightwave Technol..

[136] Urade Y., Nakata Y., Okimura K., Nakanishi T., Miyamaru F., Takeda M.W., Kitano M. (2016). Dynamically babinet-invertible metasurface: A capacitive-inductive reconfigurable filter for terahertz waves using vanadium-dioxide metal-insulator transition. Opt. Express.

[137] Nouman M.T., Hwang J.H., Faiyaz M., Lee K.J., Noh D.Y., Jang J.H. (2018). Vanadium dioxide based frequency tunable metasurface filters for realizing reconfigurable terahertz optical phase and polarization control. Opt. Express.

[138] Wang D.C., Zhang L.C., Gu Y.H., Mehmood M.Q., Gong Y.D., Srivastava A., Jian L.K., Venkatesan T., Qiu C.W., Hong M.H. (2015). Switchable ultrathin quarter-wave plate in terahertz using active phase-change metasurface. Sci. Rep..

[139] Lv T.T., Li Y.X., Ma H.F., Zhu Z., Li Z.P., Guan C.Y., Shi J.H., Zhang H., Cui T.J. (2016). Hybrid metamaterial switching for manipulating chirality based on VO_2_ phase transition. Sci. Rep..

[140] Ding F., Zhong S.M., Bozhevolnyi S.I. (2018). Vanadium dioxide integrated metasurfaces with switchable functionalities at terahertz frequencies. Adv. Opt. Mater..

[141] Lagerwall J.P.F., Scalia G. (2012). A new era for liquid crystal research: Applications of liquid crystals in soft matter nano-, bio- and microtechnology. Curr. Appl. Phys..

[142] Shrekenhamer D., Chen W.C., Padilla W.J. (2013). Liquid crystal tunable metamaterial absorber. Phys. Rev. Lett..

[143] Kowerdziej R., Parka J., Krupka J., Olifierczuk M., Nowinowski-Kruszelnicki E., Jaroszewicz L., Chojnowska O. (2013). Dielectric properties of highly anisotropic nematic liquid crystals for tunable microwave components. Appl. Phys. Lett..

[144] Altmann K., Reuter M., Garbat K., Koch M., Dabrowski R., Dierking I. (2013). Polymer stabilized liquid crystal phase shifter for terahertz waves. Opt. Express.

[145] Sasaki T., Noda K., Kawatsuki N., Ono H. (2015). Universal polarization terahertz phase controllers using randomly aligned liquid crystal cells with graphene electrodes. Opt. Lett..

[146] Hokmabadi M.P., Tareki A., Rivera E., Kung P., Lindquist R.G., Kim S.M. (2017). Investigation of tunable terahertz metamaterial perfect absorber with anisotropic dielectric liquid crystal. AIP Adv..

[147] Chikhi N., Lisitskiy M., Papari G., Tkachenko V., Andreone A. (2016). A hybrid tunable THz metadevice using a high birefringence liquid crystal. Sci Rep..

[148] Vasic B., Zografopoulos D.C., Isic G., Beccherelli R., Gajic R. (2017). Electrically tunable terahertz polarization converter based on overcoupled metal-isolator-metal metamaterials infiltrated with liquid crystals. Nanotechnology.

[149] Yin Z., Wan C., Deng G., Zheng A., Wang P., Yang Y., Gao S., Yang J., Cai F., Li Z. (2018). Fast-tunable terahertz metamaterial absorber based on polymer network liquid crystal. Appl. Sci..

[150] Zhou S., Shen Z., Kang R., Ge S., Hu W. (2018). Liquid crystal tunable dielectric metamaterial absorber in the terahertz range. Appl. Sci..

[151] Kowerdziej R., Olifierczuk M., Parka J. (2018). Thermally induced tunability of a terahertz metamaterial by using a specially designed nematic liquid crystal mixture. Opt. Express.

[152] Yang L., Fan F., Chen M., Zhang X., Chang S.-J. (2017). Active terahertz metamaterials based on liquid-crystal induced transparency and absorption. Opt. Commun..

[153] Khoo I.C. (2009). Nonlinear optics of liquid crystalline materials. Phys. Rep..

[154] Anlage S.M. (2011). The physics and applications of superconducting metamaterials. J. Opt..

[155] Gu J.Q., Singh R., Tian Z., Cao W., Xing Q.R., He M.X., Zhang J.W., Han J.G., Chen H.T., Zhang W.L. (2010). Terahertz superconductor metamaterial. Appl. Phys. Lett..

[156] Jung P., Ustinov A.V., Anlage S.M. (2014). Progress in superconducting metamaterials. Supercond. Sci. Tech..

[157] Singh R., Xiong J., Chowdhury D.R., Yang H., Azad A.K., Trugman S.A., Jia Q.X., Taylor A.J., Chen H.T., Boardman A.D., Johnson N.P., Ziolkowski R.W. (2012). Thermal and ultrafast optical tuning of ultrathin high-temperature superconducting terahertz metamaterials. Metamaterials VII.

[158] Li C., Zhang C.H., Hu G.L., Zhou G.C., Jiang S.L., Jiang C.T., Zhu G.H., Jin B.B., Kang L., Xu W.W. (2016). Electrically tunable superconducting terahertz metamaterial with low insertion loss and high switchable ratios. Appl. Phys. Lett..

[159] Wu J.B., Zhang C.H., Liang L.J., Jin B.B., Kawayama I., Murakami H., Kang L., Xu W.W., Wang H.B., Chen J. (2014). Nonlinear terahertz superconducting plasmonics. Appl. Phys. Lett..

[160] Jin B.B., Zhang C.H., Engelbrecht S., Pimenov A., Wu J.B., Xu Q.Y., Cao C.H., Chen J.A., Xu W.W., Kang L. (2010). Low loss and magnetic field-tunable superconducting terahertz metamaterial. Opt. Express.

[161] Chen H.T., Yang H., Singh R., O’Hara J.F., Azad A.K., Trugman S.A., Jia Q.X., Taylor A.J. (2010). Tuning the resonance in high-temperature superconducting terahertz metamaterials. Phys. Rev. Lett..

[162] Keller J., Scalari G., Appugliese F., Mavrona E., Rajabali S., Suess M.J., Beck M., Faist J. (2018). High T-c superconducting THz metamaterial for ultrastrong coupling in a magnetic field. ACS Photonics.

[163] Srivastava Y.K., Singh R. (2017). Impact of conductivity on lorentzian and fano resonant high-Q THz metamaterials: Superconductor, metal and perfect electric conductor. J. Appl. Phys..

[164] Han C., Li C., Wu J.B., Zhou X.J., Li J., Jin B.B., Wang H.B., Wu P.H. (2018). A study of thermal effects in superconducting terahertz modulator by low temperature scanning laser microscope. AIP Adv..

[165] Wu J.B., Jin B.B., Xue Y.H., Zhang C.H., Dai H., Zhang L.B., Cao C.H., Kang L., Xu W.W., Chen J. (2011). Tuning of superconducting niobium nitride terahertz metamaterials. Opt. Express.

[166] Wu J.B., Jin B.B., Wan J., Liang L.J., Zhang Y.G., Jia T., Cao C.H., Kang L., Xu W.W., Chen J. (2011). Superconducting terahertz metamaterials mimicking electromagnetically induced transparency. Appl. Phys. Lett..

[167] Zhang C.H., Jin B.B., Han J.G., Kawayama I., Murakami H., Wu J.B., Kang L., Chen J., Wu P.H., Tonouchi M. (2013). Terahertz nonlinear superconducting metamaterials. Appl. Phys. Lett..

[168] Ricci M., Orloff N., Anlage S.M. (2005). Superconducting metamaterials. Appl. Phys. Lett..

[169] Jaekel C., Waschke C., Roskos H.G., Kurz H. (1994). Surface-resistance and penetration depth of YBa_2_Cu_3_O_7_-_δ_ thin-films on silicon at ultrahigh frequencies. Appl. Phys. Lett..

[170] Wilke I., Khazan M., Rieck C.T., Kuzel P., Kaiser T., Jaekel C., Kurz H. (2000). Terahertz surface resistance of high temperature superconducting thin films. J. Appl. Phys..

[171] Keiser G.R., Zhang J.D., Zhao X.G., Zhang X., Averitt R. (2016). Terahertz saturable absorption in superconducting metamaterials. J. Opt. Soc. Am. B.

[172] Savinov V., Fedotov V.A., Anlage S.M., de Groot P.A.J., Zheludev N.I. (2012). Modulating sub-THz radiation with current in superconducting metamaterial. Phys. Rev. Lett..

[173] Keller J., Maissen C., Scalari G., Beck M., Cibella S., Leoni R., Faist J. (2017). Combining a fully switchable THz superconducting metamaterial with a 2DEG for ultra-strong coupling. Eur. Phys. J. Plus.

[174] Savinov V., Delfanazari K., Fedotov V.A., Zheludev N.I. (2016). Giant nonlinearity in a superconducting sub-terahertz metamaterial. Appl. Phys. Lett..

[175] Mazdouri B., Javadzadeh S.M.H. (2017). Modelling nonlinearity in superconducting split ring resonator and its effects on metamaterial structures. Phys. C-Supercond. Appl..

[176] Lazarides N., Tsironis G.P. (2007). RF superconducting quantum interference device metamaterials. Appl. Phys. Lett..

[177] Shanehsazzadeh F., Jabbari T., Qaderi F., Fardmanesh M. (2017). Integrated monolayer planar flux transformer and resonator tank circuit for High-*T_c_*RF-SQUID magnetometer. Ieee. T. Appl. Supercon.

[178] Muller M.M., Maier B., Rockstuhl C., Hochbruck M. (2019). Analytical and numerical analysis of linear and nonlinear properties of an rf-SQUID based metasurface. Phys. Rev. B.

[179] Kozlov G.V., Lebedev S.P., Mukhin A.A., Prokhorov A.S., Fedorov I.V., Balbashov A.M., Parsegov I.Y. (1993). Submillimeter backward-wave oscillator spectroscopy of the rare-earth orthoferrites. IEEE. Trans. Magn..

[180] Zhou R., Jin Z., Li G., Ma G., Cheng Z., Wang X. (2012). Terahertz magnetic field induced coherent spin precession in YFeO3. Appl. Phys. Lett..

[181] Yamaguchi K., Kurihara T., Minami Y., Nakajima M., Suemoto T. (2013). Terahertz time-domain observation of spin reorientation in orthoferrite ErFeO_3_ through magnetic free induction decay. Phys. Rev. Lett..

[182] Constable E., Cortie D.L., Horvat J., Lewis R.A., Cheng Z., Deng G., Cao S., Yuan S., Ma G. (2014). Complementary terahertz absorption and inelastic neutron study of the dynamic anisotropy contribution to zone-center spin waves in a canted antiferromagnetNdFeO_3_. Phys. Rev. B.

[183] Yamaguchi K., Nakajima M., Suemoto T. (2010). Coherent control of spin precession motion with impulsive magnetic fields of half-cycle terahertz radiation. Phys. Rev. Lett..

[184] Nakajima M., Yamaguchi K., Suemoto T. (2012). Ultrafast coherent control of spin precession motion by terahertz magnetic pulses. Acta Physica Polonica A.

[185] Jin Z., Mics Z., Ma G., Cheng Z., Bonn M., Turchinovich D. (2013). Single-pulse terahertz coherent control of spin resonance in the canted antiferromagnet YFeO_3_, mediated by dielectric anisotropy. Phys. Rev. B.

[186] Kurihara T., Nakamura K., Yamaguchi K., Sekine Y., Saito Y., Nakajima M., Oto K., Watanabe H., Suemoto T. Interactive magnetic coupling between spin precession and split-ring resonator in the terahertz frequency. Proceedings of the 2014 39th International Conference on Infrared Millimeter. Terahertz Waves (IRMMW-THz).

[187] Mukai Y., Hirori H., Yamamoto T., Kageyama H., Tanaka K. (2016). Nonlinear magnetization dynamics of antiferromagnetic spin resonance induced by intense terahertz magnetic field. New J. Phys..

[188] Kurihara T., Watanabe H., Nakajima M., Karube S., Oto K., Otani Y., Suemoto T. (2018). Macroscopic magnetization control by symmetry breaking of photoinduced spin reorientation with intense terahertz magnetic near field. Phys. Rev. Lett..

[189] Su Z.X., Zhao Q., Song K., Zhao X.P., Yin J.B. (2017). Electrically tunable metasurface based on Mie-type dielectric resonators. Sci. Rep..

